# Next-generation sequencing identifies monogenic diabetes in 16% of patients with late adolescence/adult-onset diabetes selected on a clinical basis: a cross-sectional analysis

**DOI:** 10.1186/s12916-019-1363-0

**Published:** 2019-07-11

**Authors:** Xavier Donath, Cécile Saint-Martin, Danièle Dubois-Laforgue, Ramanan Rajasingham, François Mifsud, Cécile Ciangura, José Timsit, Christine Bellanné-Chantelot, on behalf of the Monogenic Diabetes Study Group of the Société Francophone du Diabète

**Affiliations:** 1 Department of Diabetology, Cochin Hospital, Assistance Publique-Hôpitaux de Paris (AP-HP), and Paris Descartes University, DHU AUTHORS, 27 rue du Faubourg Saint-Jacques, 75014 Paris, France; 2 Department of Genetics, Pitié-Salpêtrière Hospital, AP-HP, Sorbonne University, 47/83 boulevard de l’Hôpital, 75013 Paris, France; 3 PRISIS Reference Center for Rare Diseases, Paris, France; 4 INSERM U1016, Cochin Hospital, 22 rue Méchain, 75014 Paris, France; 5 Department of Diagnostic and Interventional Radiology, and Neuroradiology, Bretonneau Hospital, University Hospital of Tours, 2 boulevard Tonnellé, 27000 Tours, France; 6 Department of Diabetology, Pitié-Salpêtrière Hospital, AP-HP, Sorbonne University, 47 Boulevard de l’Hôpital, 75013 Paris, France

**Keywords:** Monogenic diabetes, MODY, Next-generation sequencing, Molecular diagnostics, Variant of uncertain significance, Pathogenic variants

## Abstract

**Background:**

Monogenic diabetes (MgD) accounts for 1–2% of all diabetes cases. In adults, MgD is difficult to distinguish from common diabetes causes. We assessed the diagnosis rate and genetic spectrum of MgD using next-generation sequencing in patients with late adolescence/adult-onset diabetes referred for a clinical suspicion of MgD.

**Methods:**

This cross-sectional study was performed in 1564 probands recruited in 116 Endocrinology departments. Inclusion criteria were the absence of diabetes autoantibodies, and at least two of the three following criteria: an age ≤ 40 years and a body mass index (BMI) < 30 kg/m^2^ at diagnosis in the proband or in at least two relatives with diabetes, and a family history of diabetes in ≥ 2 generations. Seven genes (*GCK*, *HNF1A*, *HNF4A*, *HNF1B*, *ABCC8*, *KCNJ11*, and *INS*) were analyzed. Variant pathogenicity was assessed using current guidelines.

**Results:**

Pathogenic variants were identified in 254 patients (16.2%) and in 23.2% of EuroCaucasian patients. Using more stringent selection criteria (family history of diabetes in ≥ 3 generations, age at diabetes ≤ 40 years and BMI < 30 kg/m^2^ in the proband, EuroCaucasian origin) increased the diagnosis rate to 43%, but with 70% of the identified cases being missed. *GCK* (44%), *HNF1A* (33%), and *HNF4A* (10%) accounted for the majority of the cases. *HNF1B* (6%), *ABCC8*/*KCNJ11* (4.4%), and *INS* (2.8%) variants accounted for 13% of the cases. As compared to non-monogenic cases, a younger age, a lower BMI and the absence of diabetes symptoms at diagnosis, a EuroCaucasian origin, and a family history of diabetes in ≥ 3 generations were associated with MgD, but with wide phenotype overlaps between the two groups. In the total population, two clusters were identified, that mainly differed by the severity of diabetes at onset. MgDs were more prevalent in the milder phenotypic cluster. The phenotypes of the 59 patients (3.8%) with variants of uncertain significance were different from that of patients with pathogenic variants, but not from that of non-monogenic patients.

**Conclusion:**

Variants of *HNF1B* and the K-ATP channel genes were more frequently involved in MgD than previously reported. Phenotype overlapping makes the diagnosis of MgD difficult in adolescents/adults and underlies the benefit of NGS in clinically selected patients.

**Electronic supplementary material:**

The online version of this article (10.1186/s12916-019-1363-0) contains supplementary material, which is available to authorized users.

## Background

Monogenic diabetes, which consists mainly of maturity onset diabetes of the young (MODY), accounts for 1–2% of all diabetes cases [1]. The diagnosis of monogenic diabetes is an example of precision medicine [2, 3] because it conveys specificities as regards the severity and the course of hyperglycemia, the risk of diabetes complications, the need for diabetes treatment and its modalities, the presence of associated features, and the management of affected women during pregnancy. It also allows for familial genetic screening and counseling.

However, it has been estimated that about 50–80% of patients with MODY are either undiagnosed or misdiagnosed as type 1 or type 2 diabetes and might be inadequately treated [1, 4, 5]. In subjects with childhood or young-onset insulin-treated diabetes, recent population-based studies have shown that algorithms including the absence of markers of autoimmune type 1 diabetes [5–7] and the presence of detectable insulin secretion [7] improved differential diagnosis between type 1 diabetes and monogenic diabetes. By contrast, the diagnosis of monogenic diabetes in adults is a more complex task [8]. The heterogeneity of diabetes phenotypes in adults, the absence of diagnostic markers specific for type 2 diabetes (T2D), and the increasing prevalence of obesity in the general population and of T2D in young individuals, all make difficult differential diagnosis between monogenic and more common etiologies [9, 10].

In the recent years, next-generation sequencing (NGS) techniques, enabling the simultaneous analysis of multiple genes, have been integrated into diagnostic practice. Although more than 30 genes have been associated with monogenic diabetes [11], population studies using NGS in patients with young-onset diabetes [6] and in adults [7, 12] have consistently shown that three genes (*GCK*, *HNF1A*, and *HNF4A*) account for the large majority of MODY cases, one (*HNF1B*), associated with renal features, is less frequently involved, and three others (*ABCC8*, *KCNJ11*, and *INS*) are rare causes. Variants of other genes are either extremely rare causes of monogenic diabetes or with limited evidence of causality [6, 7, 12].

In the present study, using targeted NGS of the seven genes most frequently involved in monogenic diabetes [6, 7, 12], we analyzed a large, consecutively collected, multiethnic series of patients with adolescence or adult-onset diabetes and a clinical suspicion of monogenic diabetes. The aims of our study were (1) to assess the rate of monogenic diabetes in this population in the context of routine genetic testing, (2) to describe the frequency of monogenic diabetes subtypes when no a priori clinically driven hypothesis is made, and (3) to assess whether clinical criteria may be refined to identify patients in whom genetic screening is worth.

## Methods

### Patients

From January 2014 to October 2017, 1564 unrelated patients with a personal and/or a family history of hyperglycemia or diabetes and consecutively referred for genetic screening by 116 departments of Endocrinology and Diabetology throughout France were included in this study (Additional file 1: List of Investigators).

Selection criteria for genetic testing were the absence of type 1 diabetes-associated autoantibodies (GAD and/or IA-2, and/or ZnT8) in all participants, and at least two of the three following criteria: (1) an age at diabetes or impaired fasting glucose diagnosis ≥ 15 years and ≤ 40 years in the proband, or in at least two relatives with diabetes; (2) the absence of obesity (i.e., a body mass index (BMI) < 30 kg/m^2^) in the proband or in at least two relatives with diabetes; and (3) a family history of diabetes in at least two generations.

Patients with a family history of neonatal diabetes mellitus (NDM), hyperinsulinemic hypoglycemia of infancy, and those with a personal or a familial history suggesting HNF1B-MODY or maternally inherited diabetes and deafness were excluded from this study to avoid a recruitment bias due to these specific phenotypes.

Clinical and biological characteristics and diabetes treatment at diagnosis were recorded on standardized forms that were reviewed by three of us (XD, DDL, JT). According to the declaration of Helsinki, all patients gave written informed consent for genetic studies that included consent for the use of anonymous data for research purpose and scientific publication (CNIL certificate 1412729). All material (blood and DNA samples) were declared to French Health Authorities in compliance with current legislation.

### Genetic analyses

Genetic testing was carried out in two steps. The first one was the targeted NGS based on a multiplex PCR assay (MODY-MASTR™ assay, Agilent). The coding regions ± 30 bp of 7 genes (*GCK*, *HNF1A*, *HNF4A*, *HNF1B*, *ABCC8*, *KCNJ11*, and *INS*) and the minimal promoter region of *HNF1A*, *HNF4A*, and *INS* were amplified, and multiplex libraries were subsequently pooled and run on a MiSeq instrument (Illumina). Alignments, variant calling, and annotations were performed with the SEQNEXT software version v4.2.2 (JSI Medical Systems). All regions of interest had 100% coverage with a minimal threshold of 40 reads at each nucleotide position. Sequence variants considered as disease-causing were confirmed by Sanger sequencing.

Secondly, the search for large genomic deletions (exonic or whole-gene deletions) was performed by analyzing the genescan profiles of the multiplex reactions and, in individuals without any truncating variants identified by NGS, by the search for copy number variations in *GCK*, *HNF1A*, *HNF4A*, and *HNF1B* genes by multiplex ligation-dependent probe assay (SALSA MLPA P241 MODY, MRC-Holland).

### Variant annotation

We used the sequence variant nomenclature recommendations [13] for describing variants and classified them following the American College of Medical Genetics and Genomics (ACMG) guidelines [14]. Interpretation of sequence variants was based on the following criteria: (1) the variant type, i.e., truncating variants (nonsense, frameshift, canonical ± 1 or ± 2 splice sites, single or multi-exon deletions) vs*.* other variants (missense, in-frame variants, promoter variants); (2) functional data for reported variants (Human Gene Mutation Database [15]); (3) variant allele frequencies (VAF) in population databases (gnomeAD [16]); (4) segregation data in available pedigrees from our diagnostic database and from the literature; and (5) computational and predictive lines of evidence either suggesting an impact on gene function or predicting a pathogenic effect based on in silico analyses. For missense variants, we used 4 predictive algorithms (SIFT, PolyPhen-2, Align-GVGD, and CADD), and for splice site defects, the algorithms MaxEntScan and Splice site Finder. All algorithms, except CADD, were run with the Alamut Visual version 2.7 software (Interactive Biosoftware).

Variants were classified independently by two geneticists (CBC, CSM) into five categories: “pathogenic” (class 5), “likely pathogenic” (class 4), “uncertain significance” (class 3), “likely benign” (class 2), or “benign” (class 1), according to the ACMG rules [14]. Three groups of patients were considered: those with class 4–5 variants, those with class 3 variants, and those with no class 3–5 variants referred to as “non-monogenic” patients.

### Statistical analyses

Data are shown as medians and IQRs (interquartile ranges) or as numbers and percentages. Univariable analyses were made using non-parametric tests. Categorical variables were compared with Fisher’s exact test. Correlations were assessed by Spearman’s rank order correlation. For multivariable analyses, variables associated with a diagnosis of monogenic diabetes with a *P* value < 0.05 in the univariable analyses were included in multiple logistic regression models, and manual backward elimination procedures were performed to choose the final models. In case of collinearity between two or more variables, the most clinically pertinent was chosen. Adjusted odds ratios (ORs) were reported with their 95% confidence intervals (CI). The performance of the models to predict the diagnosis of monogenic diabetes was assessed by receiver operating characteristic (ROC) analyses. Statistical analyses were performed using GraphPad InStat (version 3.05; GraphPad Software, CA) and XLSTAT (version 2017.5, Addinsoft).

### Cluster analyses

Non-supervised hierarchical clustering was performed in R software by hclust algorithm with an average link [17]. The distance matrix between all individuals was built using a Gower metric [18], taking into account all variables, but blinded from the genetic status (monogenic or not). The optimal number of clusters was chosen based on the average silhouette width criterion [19, 20]. The population was thus parted into clusters in which the characteristics of the patients and the rates of monogenic diabetes were compared.

## Results

### Diagnosis rate and identified variants

The study included 1564 probands (827 females, 737 males) aged 15 or more at diabetes diagnosis (median 30 years, IQR 23–38). At the time of the study, the median age of the patients was 41 years [31–52], the median duration of diabetes was 6 years [1–16] and 59.2% of the patients were of EuroCaucasian origin.

**Fig. 1:**
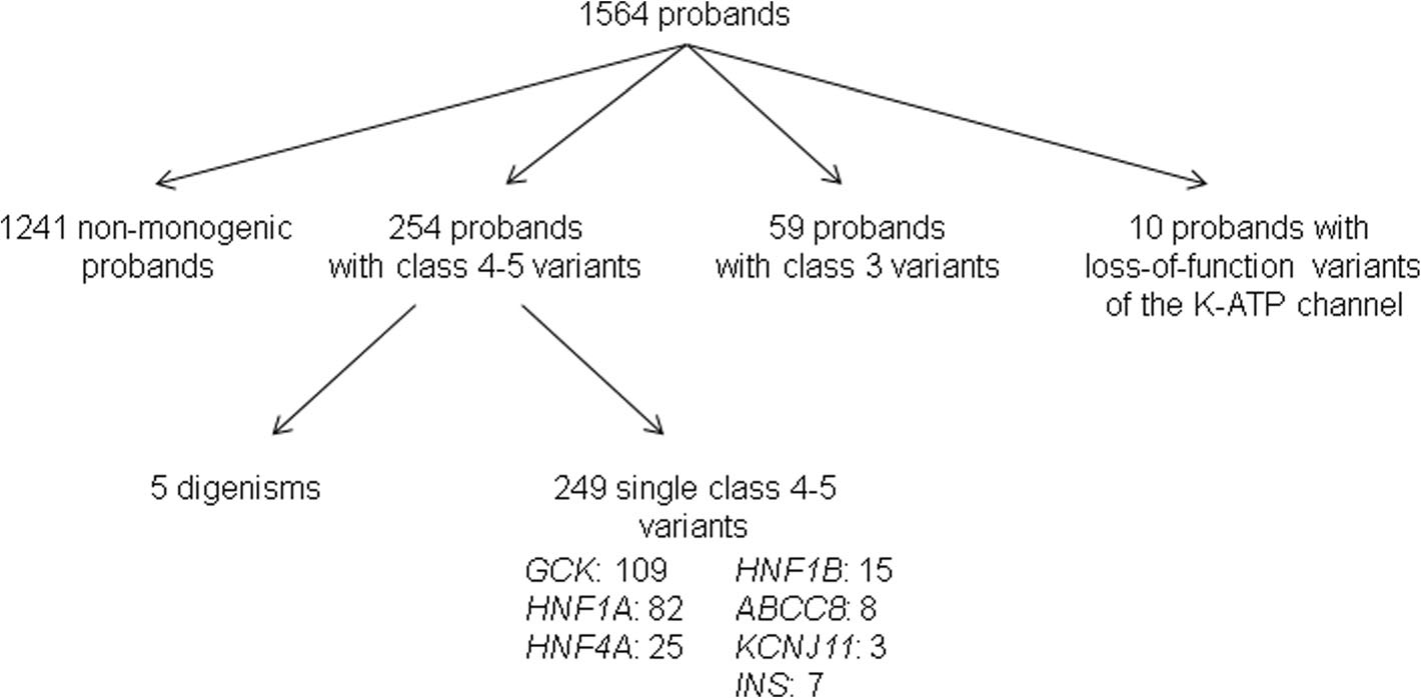
Flowchart of the next generation sequencing of seven genes in 1564 probands with diabetes

Class 4–5 variants were identified in 254 patients, leading to the diagnosis of monogenic diabetes in 16.2% of the study population (Fig. 1). In five patients, variants were found in two genes, either *GCK*/*HNF1A*, *GCK*/*ABCC8*, *GCK*/*KCNJ11*, *HNF1A*/*HNF1B*, or *HNF4A*/*ABCC8.* Their main characteristics are reported in Additional file 2: Table S1. In ten other patients, loss-of-function variants in *ABCC8* or *KCNJ11* were identified (Fig. 1 and Additional file 2: Table S2). These 10 patients were not included in the statistical analyses.

Among the 249 single class 4–5 variants, 175 were distinct, including 66 novel variants (Additional file 2: Table S3). In 216 (87%) patients, a diagnosis related to one of the three genes commonly associated with MODY (*GCK*, *HNF1A*, and *HNF4A*) was made, *GCK* accounting for 44% of all cases. In 33 (13%) patients, class 4–5 variants were identified in one of four genes usually considered as rarely involved in monogenic diabetes in adults, namely *HNF1B* (15 cases, 6.0%, among which 10 *HNF1B* whole-gene deletion), *ABCC8* (8 cases, 3.2%), *KCNJ11* (3 cases, 1.2%), and *INS* (7 cases, 2.8%) (Fig. 1, Additional file 2: Tables S3 and S4).

### Characteristics of the patients with monogenic diabetes and diagnostic criteria

The main characteristics of the 249 patients with class 4–5 variants according to genetic subtypes are shown in Table 1. In all subtypes but GCK-MODY, we observed a large scatter of patients’ characteristics, leading to a wide overlap among the different genetic subgroups (Additional file 2: Figure S1). As expected, patients with *GCK* variants (*n* = 109) had a milder phenotype than the patients with other monogenic subtypes considered as a group (*n* = 140) (not shown). As regards the 15 patients with HNF1B-MODY, renal morphology and renal function were normal in 8 of 11 and in 12 of 13 tested patients, respectively (Additional file 2 Table S5).

**Table 1:** Main characteristics at diabetes diagnosis in 249 patients with monogenic diabetes according to genetic subtype

	*GCK*	*HNF1A*	*HNF4A*	*HNF1B*	*ABCC8*	*KCNJ11*	*INS*
*N* patients	109	82	25	15	8	3	7
Sex: F/M	77/32 (71%)	57/25 (70%)	16/9 (64%)	4/11 (27%)	5/3 (63%)	2/1 (67%)	4/3 (57%)
EuroCaucasian/others (%)	90/5 (95%)	59/17 (78%)	22/1 (96%)	8/6 (57%)	5/3 (63%)	2/1 (67%)	4/3 (57%)
Age (years) (*n*)	24 [18–30] (109)	22 [18.3–27] (82)	26 [18–31] (25)	27 [18–33.5] (15)	31.5 [26.8–34.5] (8)	21 [20.5–36] (3)	22 [19–24] (7)
≥ 3 generations with diabetes: yes/no (%)	59/48 (55%)	45/34 (57%)	19/6 (76%)	7/8 (47%)	6/2 (75%)	1/2 (33%)	4/3 (57%)
BMI (kg/m^2^) (*n*)	21.1 [19.9–22.9] (95)	22 [20.1–23.8] (70)	25.1 [22.8–27.6] (25)	20.8 [19.1–23.7] (12)	22.2 [21.4–22.8] (5)	20.8 [20.4–25.1] (3)	23.9 [23.3–28.4] (7)
BMI: normal/increased (%)	85/10 (89%)	60/11 (85%)	11/14 (44%)	10/2 (83%)	4/1 (80%)	2/1 (67%)	4/3 (57%)
Symptoms of diabetes^a^: yes/no (%)	4/99 (4%)	14/63 (18%)	4/20 (17%)	11/4 (73%)	1/6 (14%)	1/2 (33%)	1/6 (14%)
HbA_1c_ (%) (*n*)	6.4 [6.2–6.6] (69)	7.9 [6.6–9.6] (45)	8.0 [6.3–9.5] (17)	10.3 [7.1–12.1] (9)	8.0 [7.5–8.6] (5)	7.4 [7.3–8.2] (3)	9 [8.2–9.7] (6)
HbA_1c_ (mmol/mol) (*n*)	46 [44–49] (69)	63 [49–81) (45)	64 [45–80] (17)	89 [54–109] (9)	64 [58–70] (5)	57 [56–66] (3)	75 [66–83] (6)
Insulin therapy: yes/no (%)	2/93 (2%)	16/57 (22%)	6/18 (25%)	11/4 (73%)	1/5 (17%)	0/3 (0%)	1/6 (14%)
Hypertension: yes/no (%)	6/62 (9%)	9/38 (19%)	4/12 (25%)	0/7 (0%)	2/5 (29%)	0/3 (0%)	0/5 (0%)
Dyslipidemia: yes/no (%)	7/52 (12%)	5/37 (12%)	4/12 (25%)	2/5 (29%)	3/2 (60%)	0/2 (0%)	2/3 (40%)

Values are actual numbers with percentages into parentheses, or median with interquartile range into brackets and numbers of values into parentheses. Patients with class 4–5 variants, excluding 5 patients with digenism, and 10 with loss-of-function variants of the K-ATP channel

*BMI* body mass index

^a^Symptoms of diabetes: polyuria and/or unexplained body weight loss and/or diabetic ketoacidosis

We compared the patients with monogenic diabetes to those with non-monogenic diabetes. In the univariable analysis, all patients’ characteristics were strongly different between the two groups (Additional file 2: Table S6). However, a large overlap was observed in the characteristics of the two groups (Additional file 2: Figure S2). Removing GCK cases from the monogenic group did not significantly alter the differences observed between monogenic and non-monogenic patients (Additional file 2: Table S6).

In the multivariable analysis, a EuroCaucasian origin, a family history of diabetes in more than two generations, a younger age, a lower BMI, and the absence of symptoms at diabetes diagnosis remained independently associated with the diagnosis of monogenic diabetes (Table 2). The area under the curve (AUC) of ROC analysis was 0.82. When GCK cases were excluded from the analysis, the same variables remained associated with the diagnosis of monogenic diabetes (Table 2), and the AUC of the model decreased to 0.79. Comparing GCK class 4–5 cases only to non-monogenic cases identified the same associations, with a higher AUC (0.89) (Table 2).

**Table 2:** Clinical characteristics associated with the diagnosis of monogenic diabetes: multivariable analysis

	Monogenic vs. non-monogenic^a^		Monogenic excluding GCK vs. non-monogenic		GCK only vs. non-monogenic	
*N*: monogenic vs. non-monogenic	194 vs. 926		113 vs.926		81 vs. 926	
Variable	*P*	OR [95% CI]	*P*	OR [95% CI]	*P*	OR [95% CI]
Sex: female vs. male	0.2866	1.22 [0.84–1.77]	0.2139	1.32 [0.85–2.05]	0.6597	1.13 [0.65–1.98]
EuroCaucasian origin: yes vs. no	< 10^−4^	3.83 [2.48–5.95]	< 10^−4^	2.70 [1.66–4.41]	< 10^−4^	9.17 [3.57–23.26]
*N* affected generations ≥ 3: yes vs. no	0.0136	1.57 [1.10–2.25]	0.0036	1.91 [1.24–2.96]	0.798	1.07 [0.63–1.81]
Age at diabetes	< 10^−4^	1.09 [1.07–1.11]	< 10^−4^	1.09 [1.06–1.11]	< 10^−4^	1.10 [1.06–1.13]
BMI at diabetes	< 10^−4^	1.13 [1.08–1.17]	0.0001	1.09 [1.04–1.14]	< 10^−4^	1.19 [1.11–1.27]
Symptoms of diabetes: yes vs. no	< 10^−4^	0.30 [0.19–0.47]	0.0036	0.48 [0.29–0.78]	< 10^−4^	0.06 [0.02–0.19]
AUC of the ROC analysis	0.82		0.79		0.89	

^a^Non-monogenic, no genetic etiology detected by targeted NGS on 7 genes

*BMI* body mass index

**Fig. 2:**
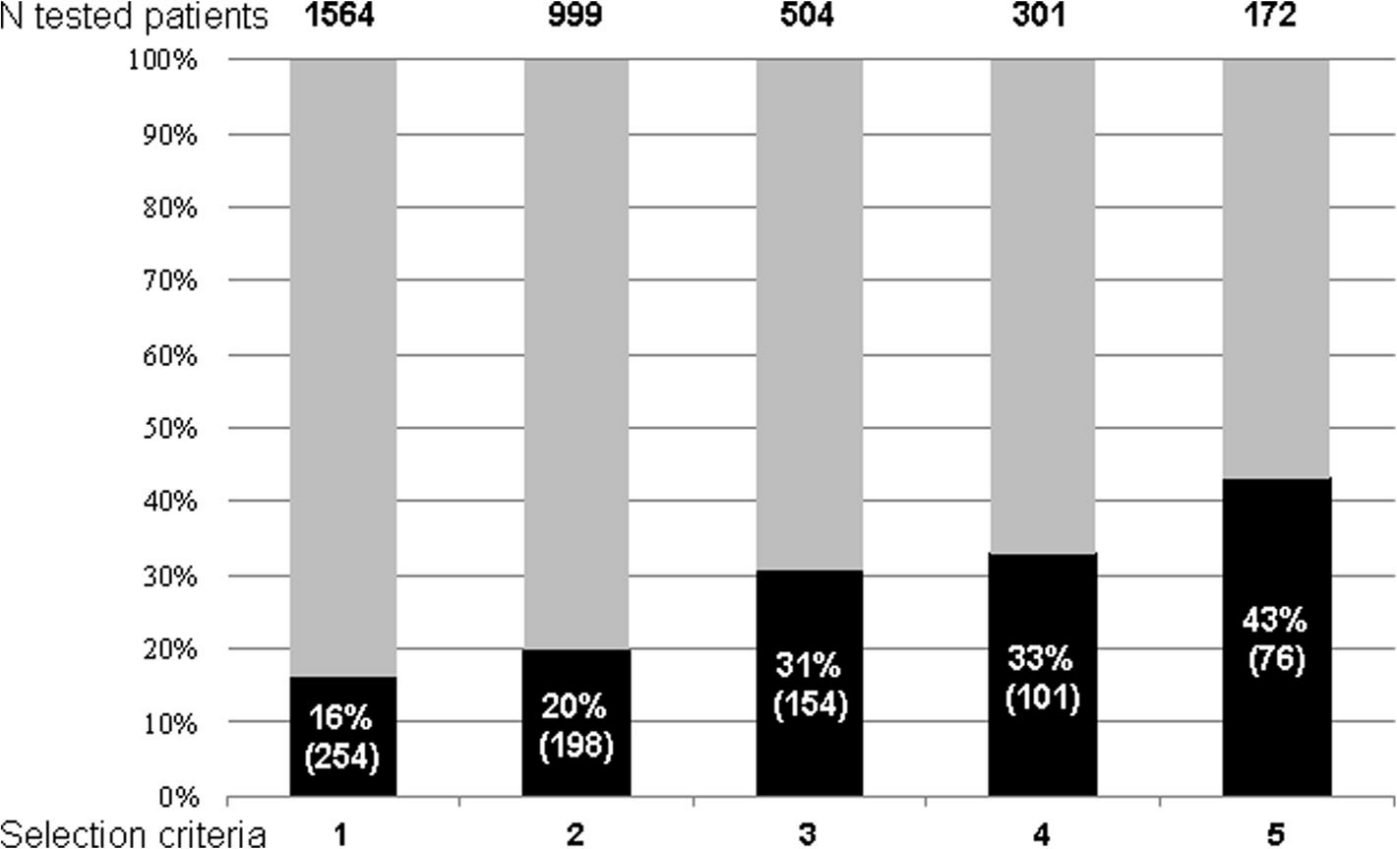
Proportions of identified monogenic cases with class 4–5 variants according to selection criteria. Actual numbers of tested patients (indicated at the top of the figure) and proportions of patients with identified monogenic diabetes (black bars, with actual numbers into parentheses) according to selection criteria. (1) Selection criteria as described in the “Methods” section; (2) patients with all 3 selection criteria; (3) patients with the 3 selection criteria plus a EuroCaucasian origin; (4) patients with a family history of diabetes in more than 2 generations, an age at diabetes diagnosis ≤ 40 years, and a body mass index < 25 kg/m^2^; and (5) same criteria as in 4 plus a EuroCaucasian origin. In total, 254 cases, i.e., 16% of the studied population, were diagnosed with monogenic diabetes. When more stringent criteria were used, the number of tested patients dramatically decreased to 11% of the total population in group 5, and the diagnostic rate increased up to 43%, but the actual number of diagnosed cases decreased sharply, 70% of the cases being missed

We assessed whether using more stringent selection criteria could improve the diagnosis rate of monogenic diabetes. As shown in Fig. 2, selecting the patients with all three selection criteria, a normal BMI at diagnosis of diabetes, and a EuroCaucasian origin would have led to perform genetic screening in only 11% of all cases and to identify monogenic diabetes in 43% of them, as compared to 16% in the total population. However, 70% of all monogenic cases of our global cohort would have been missed. In the same respect, an age at the onset of diabetes ≤ 35 years has been used to develop models intended to predict the diagnosis of monogenic diabetes [21]. In our study, using this cutoff would have led to exclude 481 patients (30.7% of the total population) from genetic testing, to increase the pick-up rate from 16.2 to 20.6% (223/1083), and to miss 12.2% (31 of the 254) of the actually identified cases.

### Diagnosis rate according to patient’s geographical origin

In our series, the frequency of class 4–5 variants was much lower in non-EuroCaucasian patients (6.5%) than in those of EuroCaucasian origin (23.2%) (*p* < 10^−4^). Patients of non-EuroCaucasian origin had a more severe diabetes phenotype than EuroCaucasians, as shown by higher HbA1c values (9.6% vs. 6.6%, *p* = 0.0001); more frequent symptoms of diabetes (44% vs. 10%, *p* < 10^−4^); and requirement for insulin therapy at diabetes diagnosis (42% vs. 13%, *p* = 0.0002). Similar differences were observed in the total population (not shown).

### Cluster analysis

**Fig. 3:**
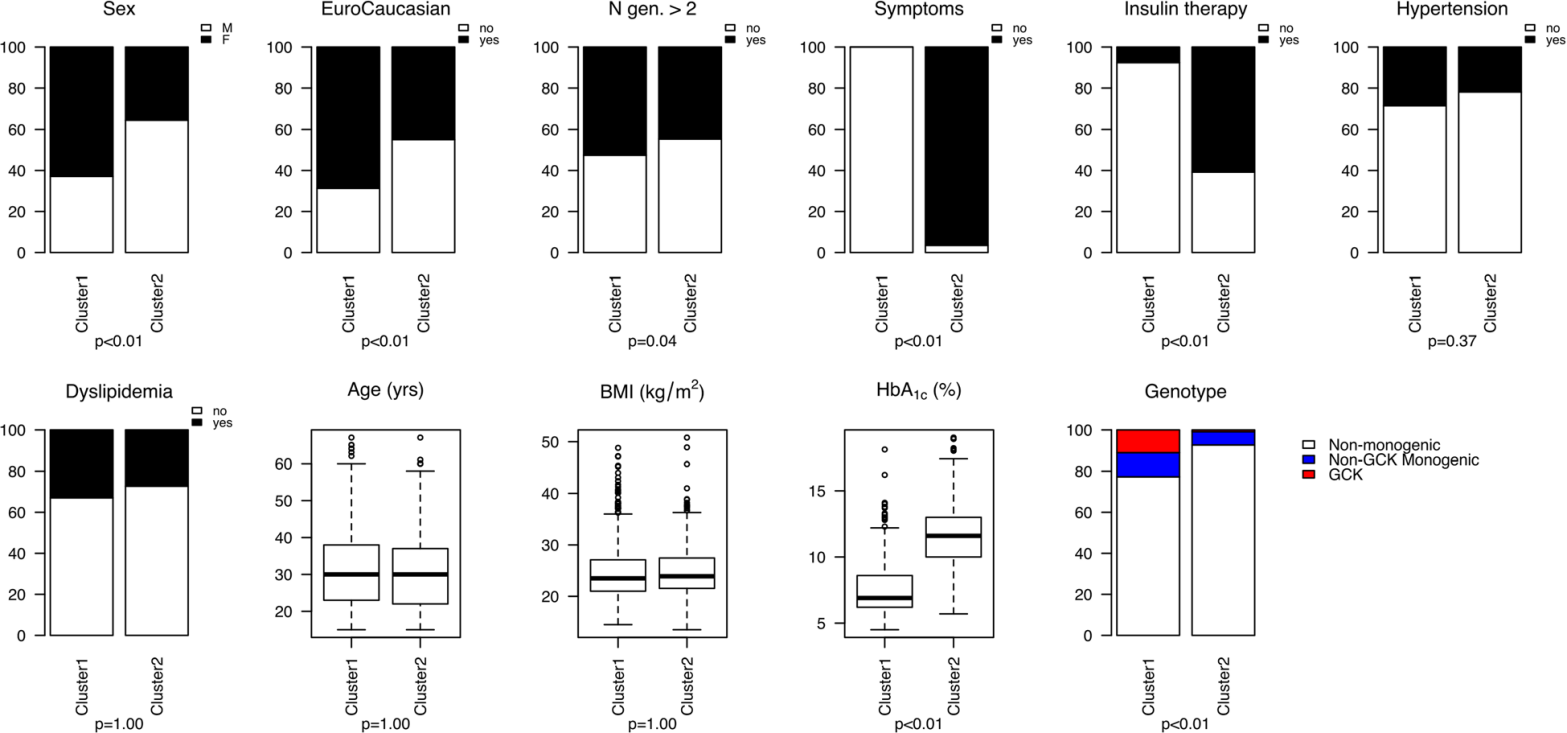
Cluster characteristics in 1495 patients with a clinical suspicion of monogenic diabetes. Among the 1495 patients, unsupervised clustering identified two clusters. Cluster 1 (937 patients) and cluster 2 (558 patients) mainly differed by the initial severity of diabetes as shown by lower frequency of clinical symptoms of diabetes and of insulin therapy and by lower HbA1c values at diabetes diagnosis. There were also more women and more subjects of EuroCaucasian origin in cluster 1. The frequency of monogenic diabetes was higher (23%) in cluster 1 than in cluster 2 (7%), with almost no GCK-MODY cases in cluster 2

To further investigate the structure of the population and the distribution of monogenic cases, unsupervised clustering was performed in 1495 patients; those with a class 3 variant of uncertain significance were excluded from this analysis. Two clusters were identified, that differed mainly by the initial severity of diabetes (Fig. 3 and Additional file 2: Figure S3). As compared to the patients in cluster 2, those in cluster 1 had no diabetes symptoms (0 of 888 vs. 498 of 516, *p* < 0.01), had a lower HbA_1c_ (7.6% vs. 11.6%, 60 mmol/mol vs. 103 mmol/mol, *p* < 0.01), and required less often insulin therapy (62 of 816 vs. 315 of 518, *p* < 0.01). They were also more often women (589 of 937 vs. 198 of 558, *p* < 0.01), more often of EuroCaucasian origin (564 of 821 vs. 228 of 507, *p* < 0.01), and had more frequently a family history of diabetes in more than 2 generations (483 of 919 vs. 242 of 542, *p* = 0.04). Monogenic cases were much more frequent in cluster 1 than in cluster 2 (23% vs. 7%, *p* < 10^−4^), with almost no GCK-MODY cases in cluster 2.

### Class 3 variants

Class 3 variants (i.e., variants of uncertain significance) were identified in 59 other patients (Fig. 1). They were mostly novel (84%) and/or missense variants (84%) for which only population frequency data and predictive in silico analyses were available (Additional file 2: Table S**7**). They were much more frequent in *ABCC8* (76%), *KCNJ11* (70%), and *INS* (30%) genes than in *GCK*, *HNF1A*, and *HNF4A* (9% of the cases, on average).

As compared to the patients with class 4–5 variants, those with class 3 variants were less often of EuroCaucasian origin (52% vs. 84%, *p* < 10^−4^), were older at diagnosis of diabetes (31 years vs. 24 years, *p* < 10^−4^), had more frequent symptoms at onset of diabetes (27% vs. 15%, *p* = 0.0484), and had higher BMI (23.7 kg/m^2^ vs. 21.8 kg/m^2^, *p* = 0.0007) and HbA_1c_ values (8.2%, vs. 6.7%, 66 mmol/mol vs. 50 mmol/mol, *p* = 0.0014). By contrast, no clinical characteristics differed between the patients with class 3 variants and those with non-monogenic diabetes (Table 3).

**Table 3:** Main characteristics of the patients with non-monogenic diabetes, class 3 variants, and class 4–5 variants

	Non-monogenic^a^	Class 3 variants	Class 4–5 variants	*p* class 3 vs. non-monogenic	*p* class 3 vs. class 4–5
*N*	1241	59	254	–	–
*N* inclusion criteria: 2/3	486/755	20/39	56/198	0.4949	0.0643
Sex: female/men, *n* (%)	619/622 (50%)	34/25 (58%)	168/86 (66%)	0.2867	0.2295
EuroCaucasian: yes/no	600/499 (55%)	28/26 (52%)	192/37 (84%)	0.7799	< 10^−4^
*N* affected generations ≥ 3, yes/no	581/631 (48%)	34/25 (58%)	144/105 (58%)	0.1818	1.0000
Age at diagnosis (years)	31 [25–39] (1239)	31 [26–37.5] (59)	24 [18–30] (254)	0.8811	< 10^−4^
BMI at diagnosis, kg/^2^ (*n*)	24.2 [21.6–27.7] (1107)	23.7 [21.7–27.3] (51)	21.8 [20.1–24.2] (221)	0.8219	0.0007
BMI: normal/increased, *n* (%)	642/475 (57%)	29/23 (56%)	180/43 (81%)	0.8861	0.0005
Symptoms at diagnosis: yes/no	461/702 (40%)	15/40 (27%)	37/204 (15%)	0.0677	0.0484
HbA_1c_ at diagnosis, % (*n*)	9.6 [7–12] (588)	8.2 [6.8–11.6] (35)	6.7 [6.3–8.7] (157)	0.4888	0.0014
HbA_1c_ at diagnosis, mmol/mol (*n*)	81 [53–108]	66 [51–103]	50 [45–72]	0.4888	0.0014
Insulin therapy: yes/no	339/768 (31%)	14/37 (27%)	38/189 (17%)	0.7560	0.1098
Arterial hypertension: yes/no	214/538 (28%)	8/32 (20%)	23/133 (15%)	0.2826	0.4667
Dyslipidemia: yes/no	235/461 (34%)	12/26 (32%)	23/116 (17%)	0.8613	0.0635

Values are actual numbers with percentages into parentheses, or median with interquartile range into brackets and numbers of values into parentheses

*BMI* body mass index

^a^Non-monogenic, no genetic etiology detected by targeted NGS on 7 genes

## Discussion

In this study, a diagnosis of monogenic diabetes was made in 16.2% of adult patients selected on clinical grounds, a better pick-up rate than that previously achieved by sequential Sanger screening, which was around 10% in adults (C. Bellanné-Chantelot, personal data).

The pick-up rate even increased to 23.2% among EuroCaucasian patients, compared to 6.5% in the patients of non-EuroCaucasian origin. This can be brought together with a study showing that the diagnosis rate of MODY was much higher among subjects of white European ethnicity than in those from non-white ethnic groups [22]. Several hypotheses can be raised, including a higher prevalence of early-onset type 2 diabetes, the involvement of other genes or oligogenic forms of diabetes in non-EuroCaucasian patients, and/or the need for population-specific screening criteria.

In keeping with previous studies [5–7, 12], class 4–5 variants of *GCK*, *HNF1A*, and *HNF4A* accounted for the large majority (87%) of monogenic diabetes diagnoses. However, the frequency of so-called rare subtypes of monogenic diabetes was unexpectedly high in patients with adult-onset diabetes. In total, *HNF1B*, *ABCC8*, *KCNJ11*, and *INS* class 4–5 variants accounted for 13% of the cases. In 15 (6%) patients, a class 4–5 *HNF1B* molecular defect was found. This was unexpected since patients had been excluded from our study when they were known to display classical phenotypes, particularly renal disease, associated with HNF1B [23]. Moreover, this 6% prevalence was higher than an estimation previously reported (< 1%) in patients with a MODY phenotype but no known renal disease [24]. Renal morphology and renal function were normal in the large majority of our HNF1B-MODY patients (Additional file S2: Table S5). Of note, among the 15 *HNF1B* cases we identified, 10 had an *HNF1B* whole gene deletion, known to be associated with a normal renal function at diabetes diagnosis in 75% of cases, but a severe diabetes phenotype [23].

We also found *ABCC8* and *KCNJ11* class 4–5 gain-of-function variants accounting for 11 (4.4%) cases. Since our study had excluded patients with a personal or a family history of NDM, it confirmed that *ABCC8*/*KCNJ11* variants can cause a milder form of diabetes that may reveal as adult-onset diabetes [25–28]. It also suggests that variants of the K-ATP channel genes may be involved in monogenic adult-onset diabetes more frequently than previously thought.

Using simple selection criteria, the diagnosis rate of monogenic diabetes was 16%, i.e., almost 10 times higher than that achieved by systematic genetic screening in adult patients with type 2 diabetes, but no MODY phenotype, diagnosed before the age of 40 years [12]. Diagnosis rate could be increased up to 43% by refining selection criteria, but at the cost of a much lower sensitivity (Fig. 2). Indeed, although almost all characteristics of the patients with monogenic vs. non-monogenic diabetes were significantly different, there were considerable overlaps between the two groups (Additional file 2: Figure S2). In the same respect, cluster analysis identified two groups of patients, with overrepresentation of monogenic diabetes cases in cluster 1, i.e., the less severe form of diabetes. However, a significant proportion of monogenic cases, as expected those with a non-GCK etiology, was observed in cluster 2. Thus, while differential diagnosis between monogenic and more common diabetes subtypes will be raised mainly in the context of adult-onset diabetes, it remains difficult to accurately select those patients in whom genetic screening is worth [8, 29].

The availability and the reducing costs of NGS technologies will theoretically allow a high-throughput sequencing of all patients with diabetes. However, one major issue is the interpretation of the results, as shown by our study: variants of uncertain significance (class 3) were identified in 59 patients, i.e., 3.8% of the total population. In the absence of functional studies, such variants should not be considered as the cause of diabetes, neither used for genetic counseling [14]. As expected, most of these variants were novel and were found in genes unfrequently studied. The clinical characteristics of the patients with class 3 variants were closer to that of the patients with non-monogenic diabetes. Whether the presence of class 3 variants should be considered as a risk factor for the occurrence of T2D or for monogenic diabetes with intermediate phenotype is still under debate [11].

Our study has several limitations. Since it was not population-based, it did not allow to calculate the prevalence of monogenic diabetes in adult patients. Rather, it was a real-life study allowing to assess the spectrum of involved genes with no a priori clinically based hypothesis on monogenic subtypes. Also, our 7-gene panel is smaller than others that included genes involved in syndromic diabetes, NDM, and insulin resistance syndromes [30–33]. Thus, one cannot exclude that rare genetic causes could have been missed. However, our panel covers nearly all non-syndromic monogenic diabetes [7, 8], and in a recent study using a much larger panel in adults with a T2D phenotype, all but one identified monogenic cases were related to these seven genes [12]. In addition, although including many genes in NGS panels is feasible, interpretation of sequence variants is complex and time consuming in a diagnostic setting, as exemplified by the high numbers of class 3 variants found in our study. As regards our selection criteria, GAD or IA-2 antibodies have been found in some patients with monogenic diabetes, but this remains a rare situation [34]. Also, in contrast with previous studies, we did not include C-peptide measurement in our selection criteria. However, those studies reported children or young individuals with insulin-requiring diabetes. In our study of adult patients, 72% did not require insulin therapy at diagnosis, indicating residual insulin secretion. Lastly, since our selection criteria included a family history of diabetes, cases with de novo variants may have been missed. However, except for *HNF1B*, de novo occurrence of pathogenic variants in the genes included in our panel remains rare [35].

## Conclusions

Our study showed that the detection rate of monogenic diabetes by NGS was high in patients with non-autoimmune adult-onset diabetes selected on simple clinical criteria. NGS also diagnosed rare monogenic diabetes subtypes in adults more frequently than previously thought. However, differential diagnosis with early-onset type 2 diabetes remained difficult.

## Additional files

Additional file 1: List of investigators of the French Monogenic Diabetes Study Group of the Société Francophone du Diabète (29 ko). (DOCX 24 kb)

Additional file 2: **Table S1.** Cases with class 3/4/5 variants identified in two genes. **Table S2.** Loss-of-function variants identified in *ABCC8* and *KCNJ11*. **Table S3.** List of novel pathogenic (Class 5) or likely pathogenic (Class 4) variants. **Table S4.** List of known class 4–5 variants identified in *ABCC8*, *HNF1B*, *KCNJ11*, and *INS* genes. **Table S5.** Main characteristics of the 15 patients with HNF1B-MODY. **Table S6.** Main characteristics at the onset of diabetes in patients with monogenic vs. non-monogenic diabetes. **Table S7.** List of variants of uncertain significance (class 3). **Figure S1.** Main characteristics at diagnosis of diabetes in patients with monogenic diabetes according to the involved gene. **Figure S2.** Main characteristics at diagnosis of diabetes in patients with (M+) and without (M−) monogenic diabetes. **Figure S3**. Hierarchical clustering of 1495 patients with a clinical suspicion of monogenic diabetes. (304 ko). (DOCX 290 kb)

## Data Availability

The datasets analyzed during the current study are available from the corresponding author on reasonable request.

## Abbreviations

ACMG: American College of Medical Genetics and Genomics
BMI: Body mass index
MODY: Maturity-onset diabetes of the young
NDM: Neonatal diabetes mellitus
NGS: Next-generation sequencing
T2D: Type 2 diabetes
