# Supplementary material for: Next-generation sequencing identifies monogenic diabetes in 16% of patients with late adolescence/adult-onset diabetes selected on a clinical basis: a cross-sectional analysis
Source: BMC Med. 2019 Jul 11;17:132. doi: 10.1186/s12916-019-1363-0 (PMC6621990; doi:10.1186/s12916-019-1363-0)
Supplement: Supplementary file 1 — List of investigators of the French Monogenic Diabetes Study Group of the Société Francophone du Diabète (29 ko). (DOCX 24 kb) [file 12916_2019_1363_MOESM1_ESM.docx]

**“Clinically-driven next-generation sequencing identifies monogenic diabetes in 16% of patients with adult-onset diabetes”: a cross-sectional analysis by Xavier Donath *et al*.**

**Additional file 1.** List of Investigators of the French Monogenic Diabetes Study Group of the Société Francophone du Diabète

**List of Investigators of the French Monogenic Diabetes Study Group of the Société Francophone du Diabète**

DELENNE B, MORLET-BARLA N, SEJIL S (CH d’Aix en Provence); COPPIN M, DESAILLOUD R, SARAVALL-GROSS M, THEBERT J (CHU Amiens); ALLIX I, BONNEAU D, GAUTHIER A, JOLLY M, LALMI F, (CHU Angers); CARREAU A, MOUTEL S, WRIGHT D, YANISSE D (CH Annecy); GUEDJ A-M (CH J Imbert, Arles); BOUROUINA A (Hôpital privé Athis Mons); AMBONVILLE C (CHIC R Ballanger, Aulnay sous Bois); GABREAU T (CH d'Auxerre); D'ANELLA P, KADEM M, LATIL-PLAT F, VILLON L, (CH d'Avignon); JAN P (CH Bar le Duc); ROUSSEAU E (CH Bayeux); DEMARSY D, MAURY E, RITZ-QUILLACQ L (CH la Côte Basque, Bayonne); OREJUELA C, (CH Belfort); ARBEY A-S, CREPIN T, HERGOTT FAURE L, SCHILLO F (CHU Besancon); LESIRE V (CH Blois); REACH G (AP-HP, Hôpital Avicennes, Bobigny); COSSON E, SITBON M (AP-HP Hôpital Jean Verdier, Bondy); DUCORNET B (AP-HP, Hôpital Ambroise Paré, Boulogne-Billancourt); FOURNIER S, LEPAGE M (CH Boulogne-sur-Mer); CROUZEIX G, JAGOT M, KERLAN V, ROUDAUT N, SONNET E, THUILLIER P (Hôpital La Cavale Blanche, Brest); LEJEUNE M, SIMOES A (CH Bligny, Briis sous Forge); DOYE E (HCL, Bron); GERARD M, HADDOUCHE A (CHU Caen); OLIVIER F (CH de Cahors); DEPARADE C (CH Carpentras); ALRAMMAH F, CAMPINOS C (CH René Dubos, Cergy-Pontoise); DU BOULLAY H, GUEDEL D, LERICHE A, WATERLOT C (CH Chambery); FAVRIEL-TRULEA M (CH Charleville-Mezières); DURIEZ T (CH de Cholet); BATISSE M, CORNELIS F, MIOLANE-DEBOUIT M, TERRAL D (CHU Clermont Ferrand); JUSTINIEN E (CH Compiègne); BERTOIN F, SOMERS F (CH Alpes Leman, Contamine sur Arve); CHARPENTIER G, DEBURGE A, HUYNH P, PENFORNIS A, REQUEDA E (CH Sud Francilien, Corbeil Essonnes); JEANNE S (CH Laennec, Creil); COURBEBAISSE B (AP-HP Hôpital Henri Mondor, Créteil); BERTRON A, MARTIN-CHAUFFIER D (CH Dieppe); BAILLOT-RUDONI S, BRINDISI M-C, CREVISY E, FOURMONT C, VERGES B, (CHU Dijon); JOANNINIS S (CH Sud Essonnes, Etampes); MULLER M, WION N (CHU Grenoble); BURES C, LAYET V (CH du Havre); SOLMON C (CH Hyères); AISSA Y (CH Langres); CLAVEL S (Hôpital Hôtel Dieu, Le Creusot); AFFRES-GRATEAU H, BOUGNERES P, CASTELL A-L, DELIVET S, KAMENICKY P, KUHN E, MAVROMATI M, SALENAVE S (AP-HP Hôpital Le Kremlin-Bicêtre);; BEROUD V, GALINAT S (CHU Limoges); DUCROCQ S (CH Lonjumeau); BOURGON C, MOUGEL F (CH de Lons Le Saunier); PERLEMOINE C (CH Lorient); ATLAN C, DADOUN F (CH de Luxembourg); LE BERRE J-P (Clinique mutualiste de Lyon) ; REFFET S (Hôpital Edouard Herriot, Lyon); BELIARD-LASSERRE S, CASTINETTI F, DOULLAY F, DUBOIS N, DUFAITRE-PATOURAUX L, GABORIT B, JANNOT-LAMOTTE M-F, MOUROUX D, RACCAH D, VALERO R, ZEVACCO-MATTEI C (APHM, Hôpital de la Timone, Marseille); COHEN J (Hôpital Saint-Joseph, Marseille) ; COHEN-VALENSI R (CH Martigues); BITU J (Hôpital Privé Jacques Cartier, Massy); MATALLAH N (CH Meaux) ; CERF-BARON I (CH Marc Jacquet, Melun); MOREAU-LE DROGUENE C, ROUGEOREILLE C (CH Melun); DEMANGE J, LOUIS J (CHU Metz); PERICHON I (CH Mont de Marsan); POUSSIER A (CH Montelimar); FLAMENT S, KASAWAT F, LE LUYER B (Hôpital Jacques Monod, Montivilliers); LEPRIEUR E, WOJTUSCISZYN A (CHU Montpellier); BARDY-BRUNET F (CH Moulins-Yzeure, Moulins); ARBEY A-S (CH Mulhouse) ; CHAILLOUS L, COLOMBEL A, FERRON-BERNAT S, LE BRAS M (CHU Nantes); CHEVALIER N, HIERONIMUS S (Hôpital de l’Archet, Nice); CHIESA J, GILLY O, GUEDJ A-M, VERIER A-C (CHRU Nimes); BRUZEAU J, COLLIN P, LEFORT G, RODES M-L, (CH Niort); KROMPA K, MATALLAH N, POTIER L, ROUSSEL R (AP-HP Hôpital Bichat, Paris); CORNU E, DEGHANI L, ELGRABLY F, FAUCHER P, LARGER E, LEMOINE A, MBEMBA J, SAMAKE M (AP-HP Hôpital Cochin, Paris); DUCLOUX R (AP-HP, Hôpital Européen Georges Pompidou, Paris); BAZ B-R, GAUTIER J-F, MEAS T, LEBLANC H, VIDAL-TRECAN T (AP-HP Hôpital Lariboisière, Paris); ARON-WISNEWSKY J, BRUCKERT E, HALBRON M, HARTEMANN A, JACQUEMINET-NEVANT S, LECORNET-SOKOL E, TOUATI E-B, VESCO L (AP-HP Hôpital La Pitié-Salpêtrière, Paris); FELDMAN-BILLARD S (CHNO des Quinze-Vingts, Paris); AMOUYAL M, CAREL J-C, MARTINERIE L, POIDVIN A, TUBIANA N (AP-HP Hôpital Robert Debré, Paris); BOURCIGAUX N, BRICAIRE L, CHRISTIN-MAITRE S, DONADILLE B, LAUTRIDOU C, VATIER C (AP-HP Hôpital Saint-Antoine, Paris); OLIVIER M-A (Hôpital Saint-Joseph, Paris) ; TEYNIE J (CH Pau) ; EID C, RADAOUI A (CH Perpignan) ; CHAUSSADE C, COFFIN-BOUTREUX C, (CH Périgueux); MAURY E (CHU Bordeaux, Hôpital Haut Lévêque, Pessac); BOURGEOIS S, CRISTINI P (CHU LYON, Hôpital Lyon Sud, Pierre-Benite); KURY-PAULIN S (CH Pontarliers) ; LAUNAY P (Clinique de l’Europe, Le Port Marly); BLANCHARD P, COSSEC A (CH de Cornouailles, Quimper); GEAMANU M (CH Rambouillet); DECOUDIER B, DELEMER B, FRANCOIS M, GAILLARD D, LUKAS- CROISIER C (CHU Reims); DERRIEN C, ESVANT A, SAADE M-B (CHU Rennes); BENOIT-TRICAUD I, DUCET-BOIFFARD A, FEIGEL-GUILLER B, HAWKEN C (CHD Vendée, La Roche sur Yon); ABDI M-L (Hôpital Drome Nord, Romans sur Isère); DURAND-GONZALEZ K, SAVOURE A (CHU Rouen); PRIOU-GUESDON M (CH Saint Brieuc); CHICHE F, COHEN R (CH de Saint Denis); APPAVOUPOULLE V, BOYER M-C, PARENTELLI A-S (CHR La Réunion, Saint Denis La Réunion); BLANC P, PERSONNIER C (CHI Poissy, Saint Germain en Laye); ANCELLE D (CH Mémorial France Etats Unis, Saint Lo); RASANDRATANA A (CH Saint Malo); BLAIZOT I, BRUNETIERE C, JACQUIN V, SUPLY O (CH de Saint Nazaire); COGNE M (CHU de la Réunion, Saint-Pierre La Réunion); GRILLOT S, YAHIA A, (CH de Sallanches); GIRARD-LEMAIRE F, JEANDIDIER N, KESSLER L, MOREAU F, ORTEGA F, PERRIN P, SCHAEFER E, SPIZZO A-H (CHU Strasbourg); VIGNON F, (CH Saint Vincent de Paul, Strasbourg); BOUILLOUD F (Hôpital Foch, Suresnes); ARRIVE J (CH de Bigorre, Tarbes); MALVAUX S (CHU Metz-Thionville, Thionville); LIOGER B (CHRU Tours); HELAN V, RIAUX F (CH Louis Domergue, La Trinité); HAULOT J-P (CH Tulle); BOHME P, JELLIMANN S, DUCHESNE L (CHU Nancy, Vandoeuvre les Nancy); ARNAULT G, PERDU S (CH Bretagne-Atlantique, Vannes); LEGER-GUIST'HAU J, SOMDA F (CH Vichy Jacques Lacarin, Vichy).
