# Supplementary material for: Next-generation sequencing identifies monogenic diabetes in 16% of patients with late adolescence/adult-onset diabetes selected on a clinical basis: a cross-sectional analysis
Source: BMC Med. 2019 Jul 11;17:132. doi: 10.1186/s12916-019-1363-0 (PMC6621990; doi:10.1186/s12916-019-1363-0)
Supplement: Supplementary file 2 — Table S1. Cases with class 3/4/5 variants identified in two genes. Table S2. Loss-of-function variants identified in ABCC8 and KCNJ11. Table S3. List of novel pathogenic (Class 5) or likely pathogenic (Class 4) variants. Table S4. List of known class 4–5 variants identified in ABCC8, HNF1B, KCNJ11, and INS genes. Table S5. Main characteristics of the 15 patients with HNF1B-MODY. Table S6. Main characteristics at the onset of diabetes in patients with monogenic vs. non-monogenic diabetes. Table S7. List of variants of uncertain significance (class 3). Figure S1. Main characteristics at diagnosis of diabetes in patients with monogenic diabetes according to the involved gene. Figure S2. Main characteristics at diagnosis of diabetes in patients with (M+) and without (M−) monogenic diabetes. Figure S3. Hierarchical clustering of 1495 patients with a clinical suspicion of monogenic diabetes. (304 ko). (DOCX 290 kb) [file 12916_2019_1363_MOESM2_ESM.docx]

**"Next-generation sequencing identifies monogenic diabetes in 16% of patients with late adolescence or adult-onset diabetes selected on a clinical basis: a cross-sectional analysis ", by Xavier Donath *et al*.**

**Additional file 2.**

- **Tables S1, S2, S3, S4, S5, S6, S7**
- **Figures S1, S2, S3**

**Table S1.** Cases with class 3/4/5 variants identified in two genes

| **case #** | **Gene** | **Location** | **Nucleotide change** | **Protein effect** | **Variant type** | **Class of pathogenicity** | **References** | **sex** | **Euro-Caucasian** | **n of generations with diabetes** | **age (years)** | **BMI (kg/m²)** | **symptoms** | **HbA1c (%)** | **insulin therapy** |
| --- | --- | --- | --- | --- | --- | --- | --- | --- | --- | --- | --- | --- | --- | --- | --- |
| 1 | *HNF4A* | Exon 8 | c.1063G>C | p.Gly355Arg | Splice defect | 5 | This report | F | yes | 3 | 17 | 32.6 | yes | na | yes |
|  | *ABCC8* | Exon 10 | c.1561C>T | p.Arg521Trp | Misense | 3 | This report |  |  |  |  |  |  |  |  |
| 2 | *GCK* | Exon 10 | c.1333A>T | p.Ser445Cys | Missense | 4 | [1] | M | yes | 4 | 20 | 23.8 | no | 7.5 | no |
|  | *KCNJ11* | Exon 1 | c.1040G>A | p.Arg347His | Missense | 3 | [2] |  |  |  |  |  |  |  |  |
| 3 | *GCK* | Exon 2 | c.128G>A | p.Arg43His | Missense | 5 | [3] | F | na | 1 | 38 | 20.8 | no | 6.1 | na |
|  | *HNF1A* | Exon 4 | c.862G>A | p.Gly288Arg | Missense | 3 | This report |  |  |  |  |  |  |  |  |
| 4 | *HNF1A* | Exon 4 | c.872dupC | p.Gly292fs | Frameshift | 5 | [4] | M | no | 4 | 37 | 21.1 | no | 6.2 | no |
|  | *HNF1B* | Exon 3 | c.793G>A | p.Val265Met | Missense | 3 | This report |  |  |  |  |  |  |  |  |
| 5 | *GCK* | Exon 5 | c.571C>T | p.Arg191Trp | Missense | 5 | [1] | F | na | 2 | 20 | na | no | na | no |
|  | *ABCC8* | Exon 33 | c.4058G>C | p.Arg1353Pro | Missense | 4 | [5] |  |  |  |  |  |  |  |  |

Sequence variants are numbered with respect to GenBank cDNA sequences. *ABCC8*, NM_000352.3; *GCK*, NM_000162.3; *HNF1A*, NM_000545.6; *HNF1B*, NM_000458.3; *HNF4A*, NM_175914.4; *KCNJ11*, NM_000525.3 and described according to Human Genome Variation Society (HGVS) guidelines (<http://www.hgvs.org/varnomen>). Variants were classified according to ACMG recommendations [see Table S3 for details]. LOF, loss-of-function variants

1. Osbak KK, Colclough K, Saint-Martin C, Beer NL, Bellanné-Chantelot C, Ellard S, Gloyn AL.: **Update on mutations in glucokinase (GCK), which cause maturity-onset diabetes of the young, permanent neonatal diabetes, and hyperinsulinemic hypoglycemia.** *Hum Mut* 2012, **30**(11): 1512-26.

2. Kapoor RR, Flanagan SE, Arya VB, Shield JP, Ellard S, Hussain K.: **Clinical and molecular characterisation of 300 patients with congenital hyperinsulinism.** *Eur J Endocrinol* 2013, **168**(4): 557-64.

3. Beer NL, Osbak KK, van de Bunt M, Tribble ND, Steele AM, Wensley KJ, Edghill EL, Colcough K, Barrett A, Valentínová L, *et al*: **Insights into the pathogenicity of rare missense GCK variants from the identification and functional characterization of compound heterozygous and double mutations inherited in cis.** *Diabetes Care* 2012, **35**(7): 1482-4.

4. Colclough K, Bellanne-Chantelot C, Saint-Martin C, Flanagan SE, Ellard S.: **Mutations in the genes encoding the transcription factors hepatocyte nuclear factor 1 alpha and 4 alpha in maturity-onset diabetes of the young and hyperinsulinemic hypoglycemia.** *Hum Mut* 2013, **34**(5): 669-85.

5. Magge SN, Shyng SL, MacMullen C, Steinkrauss L, Ganguly A, Katz LE, Stanley CA.: **Familial leucine-sensitive hypoglycemia of infancy due to a dominant mutation of the beta-cell sulfonylurea receptor.** *J Clin Endocrinol Metab* 2004, **89**(9): 4450-6.

**Table S2.** Loss-of-function variants identified in *ABCC8* and *KCNJ11*

| **Gene** | **Location** | **Nucleotide Change** | **Protein effect** | **Variant type** | **Class of pathogenicity** | **References** |
| --- | --- | --- | --- | --- | --- | --- |
| *ABCC8* | Exon 2 | c.220C>T | p.Arg74Trp | Missense | 4 | (1) |
| *ABCC8*  *ABCC8* | Exon 6  Exon 10 | c.892C>T  c.1484G>A | p. Arg298Cys  p.Arg495Gln | Misense  Missense | 3  4 | (2)  (3) |
| *ABCC8*  *ABCC8* | Exon 12  Exon 18 | c.1741T>A  c.2294+1G>A | p.Ser581Thr  p.? | Missense  Splice defect | 3  5 | (4)  This report |
| *ABCC8* | Exon 35 | c.4262G>A | p.Arg1421His | Missense | 4 | (5) |
| *KCNJ11*  *KCNJ11* | 5’UTR  Exon 1 | c.-54C>T  c.148C>T | p.0?  p.Arg50Trp | Promoter variant  Missense | 3  4 | (6)  This report * |
| *KCNJ11* | Exon 1 | c.617G>A | p.Arg206His | Missense | 4 | This report * |
| *KCNJ11* | Exon 1 | c.934G>T | p.Gly312Cys | Missense | 4 | (7) |

*variant previously identified in cases diagnosed with recessive form of HHI in the diagnostic’s database of Pitié-Salpêtrière hospital

Sequence variants are numbered with respect to GenBank cDNA sequences. *ABCC8*, NM_000352.3; *KCNJ11*, NM_000525.3 and described according to Human Genome Variation Society (HGVS) guidelines (<http://www.hgvs.org/varnomen>). Variants were classified according to ACMG recommendations [see Table S3 for details].

1. Suchi M, MacMullen C, Thornton P, Adzick N, Ganguly A, Ruchelli E, Stanley C.: **Molecular and immunohistochemical analyses of the focal form of congenital hyperinsulinism.** *Mod Pathol* 2006, **19**(1): 122-9.

2. Snider KE, Becker S, Boyajian L, Shyng SL, MacMullen C, Hughes N, Ganapathy K, Bhatti T, Stanley CA, Ganguly A.: **Genotype and phenotype correlations in 417 children with congenital hyperinsulinism.** *J Clin Endocrinol Metab* 2013, **98**(2): E355-363.

3. Yan F, Lin Y, MacMullen C, Ganguly A, Stanley C, Shyng S: **Congenital hyperinsulinism associated ABCC8 mutations that cause defective trafficking of ATP-sensitive K+ channels: identification and rescue.** *Diabetes* 2007, **56**(9): 2239-48.

4. Salisbury RJ, Han B, Jennings RE, Berry AA, Stevens A, Mohamed Z, Sugden SA, De Krijger R, Cross SE, Johnson PP, *et al*.: **Altered Phenotype of beta-Cells and Other Pancreatic Cell Lineages in Patients With Diffuse Congenital Hyperinsulinism in Infancy Caused by Mutations in the ATP-Sensitive K-Channel.** *Diabetes* 2015, **64**(9): 3182-3188.

5. Saito-Hakoda A, Yorifuji T, Kanno J, Kure S, Fujiwara I.: **Nateglinide is Effective for Diabetes Mellitus with Reactive Hypoglycemia in a Child with a Compound Heterozygous ABCC8 Mutation.** *Clin Pediatr Endocrinol* 2012, **21**(3): 45-52.

6. Huopio H, Jaaskelainen J, Komulainen J, Miettinen R, Karkkainen P, Laakso M, Tapanainen P, Voutilainen R, Otonkoski T: **Acute insulin response tests for the differential diagnosis of congenital hyperinsulinism.** *J Clin Endocrinol Metab* 2002, **87**(10): 4502-4507.

7. Arya VB, Guemes M, Nessa A, Alam S, Shah P, Gilbert C, Senniappan S, Flanagan SE, Ellard S, Hussain K.: **Clinical and histological heterogeneity of congenital hyperinsulinism due to paternally inherited heterozygous ABCC8/KCNJ11 mutations .** *Eur J Endocrinol* 2014, **171**(6): 685-95.

**Table S3.** List of novel pathogenic (Class 5) or likely pathogenic (Class 4) variants

|  |  |  |  | Criteria for classifying variants according to ACMG guidelines (1) | | | | | | |  |
| --- | --- | --- | --- | --- | --- | --- | --- | --- | --- | --- | --- |
| Gene | Location | Nucleotide Change | Protein effect | Variant type | Variant consequence | Functional data | Population data | Segregation data | Computational and predictive evidence | Class of pathogenicity | |
| *ABCC8* | Exon 22 | c.2588A>G | p.His863Arg | Missense |  |  | PM2 | PP1 | PM5 PP3 | 4 | |
| *ABCC8* | Exon 37 | c.4514T>C | p.Met1505Thr | Missense |  | PS3 | PM2 |  | PP3 | 4 | |
| *GCK* | Intron 1 | c.46-1G>T | p.? | Splice defect | PVS1 |  | PM2 |  |  | 5 | |
| *GCK* | Exon 2 | c.208G>C | p.Glu70Gln | Splice defect |  | PS3 | PM2 | PP1 | PM5 PP3 PP2 | 5 | |
| *GCK* | Exon 2-3 | c.46-?_363+?del | p.? | In-frame exonic deletion | PM4 |  | PM2 |  |  | 4 | |
| *GCK* | Exon 3 | c.236T>C | p.Leu79Pro | Missense |  |  | PM2 | PS2 | PP3 PP2 | 4 | |
| *GCK* | Exon 3 | c.247A>G | p.Asn83Asp | Missense |  |  | PM2 |  | PP3 PP2 | 4 | |
| *GCK* | Exon 3 | c.268A>T | p.Lys90Ter | Nonsense | PVS1 |  | PM2 |  |  | 5 | |
| *GCK* | Exon 4 | c.388A>G | p.Ile130Val | Missense |  |  | PM2 |  | PM5 PP3 PP2 | 4 | |
| *GCK* | Exon 5 | c.485G>C | p.Gly162Ala | Missense |  |  | PM2 |  | PM5 PP3 PP2 | 4 | |
| *GCK* | Exon 5 | c.505A>G | p.Lys169Glu | Missense |  |  | PM2 |  | PM5 PP3 PP2 | 4 | |
| *GCK* | Exon 5 | c.508G>T | p.Gly170Cys | Missense |  |  | PM2 |  | PM5 PP3 PP2 | 4 | |
| *GCK* | Exon 5 | c.513C>G | p.Phe171Leu | Missense | PS1 |  | PM2 |  | PM5 PP3 PP2 | 5 | |
| *GCK* | Exon 6 | c.677T>G | p.Val226Gly | Missense |  |  | PM2 |  | PM1 PP3 PP2 | 4 | |
| *GCK* | Exon 7 | c.686del | p.Gly229fs | Frameshift | PVS1 |  | PM2 |  |  | 5 | |
| *GCK* | Exon 7 | c.770G>A | p.Trp257Ter | Nonsense | PVS1 |  | PM2 |  |  | 5 | |
| *GCK* | Exon 7 | c.781G>C | p.Gly261Arg | Missense | PS1 |  | PM2 | PP1 | PM1 PM5 PP3 PP2 | 5 | |
| *GCK* | Exon 7 | c.821A>C | p.Asp274Ala | Missense |  |  | PM2 |  | PM5 PP3 PP2 | 4 | |
| *GCK* | Exon 7 | c.824_850del | p.Arg275_Asn283del | In-frame deletion | PM4 |  | PM2 |  | PP3 | 4 | |
| *GCK* | Exon 7 | c.859C>T | p.Gln287Ter | Nonsense | PVS1 |  | PM2 |  |  | 5 | |
| *GCK* | Exon 8 | c.868G>A | p.Glu290Lys | Missense |  |  | PM2 |  | PM5 PP3 PP2 | 4 | |
| *GCK* | Exon 8 | c.904G>T | p.Val302Leu | Missense | PS1 |  | PM2 |  | PM5 PP3 PP2 | 5 | |
| *GCK* | Exon 8 | c.908G>C | p.Arg303Pro | Missense |  |  | PM2 |  | PM1 PP3 PP2 | 4 | |
| *GCK* | Exon 8 | c.1019G>A | p.Ser340Asn | Splice defect | PVS1 |  | PM2 |  | PM5 PP3 PP2 | 5 | |
| *GCK* | Exon 9 | c.1134_1151del | p.Ala379_Ala384del | In-frame deletion | PM4 |  | PM2 |  | PP3 | 4 | |
| *GCK* | Exon 9 | c.1135G>A | p.Ala379Thr | Missense |  |  | PM2 |  | PM5 PP3 PP2 | 4 | |
| *GCK* | Exon 9 | c.1181_1182delinsCG | p.Arg394Pro | Missense |  | PS3 | PM2 |  | PP3 PP2 | 4 | |
| *GCK* | Exon 9 | c.1238_1253+14delinsGCCCCCA | p.Tyr413_Ser418delinsCysProHis | Splice defect | PVS1 |  | PM2 | PP1 | PP3 | 5 | |
| *GCK* | Exon 9 | c.1246C>T | p.His416Tyr | Missense |  |  | PM2 |  | PM5 PP3 PP2 | 4 | |
| *GCK* | Exon 10 | c.1313T>C | p.Phe438Ser | Missense |  |  | PM2 |  | PM5 PP3 PP2 | 4 | |
| *GCK* | Exon 10 | c.1348del | p.Ala450fs | Frameshift | PVS1 |  | PM2 |  |  | 5 | |
| *HNF1A* | Exon 1 | c.26A>G | p.Gln9Arg | Missense |  |  | PM2 |  | PM1 PM5 PP3 | 4 | |
| *HNF1A* | Exon 1 | c.35T>G | p.Leu12Arg | Missense |  |  | PM2 |  | PM5 PP3 | 4 | |
| *HNF1A* | Exon 1 | c.102del | p.Tyr36fs | Frameshift | PVS1 |  | PM2 |  |  | 5 | |
| *HNF1A* | Exon 1 | c.142dup | p.Glu48fs | Frameshift | PVS1 |  | PM2 |  |  | 5 | |
| *HNF1A* | Exon 1 | c.242del | p.Phe81fs | Frameshift | PVS1 |  | PM2 |  |  | 5 | |
| *HNF1A* | Intron 1 | c.326+3_326+9del | p.? | Splice defect | PVS1 |  | PM2 |  | PP3 | 4 | |
| *HNF1A* | Intron 1 | c.326+4A>G | p.? | Splice defect | PVS1 |  | PM2 |  | PP3 | 4 | |
| *HNF1A* | Exon 2 | c.364T>C | p.Tyr122His | Missense |  |  | PM2 |  | PM1 PP3 | 4 | |
| *HNF1A* | Exon 2 | c.375G>C | p.Gln125His | Missense |  |  | PM2 |  | PM1 PP3 | 4 | |
| *HNF1A* | Exon 2 | c.467C>T | p.Thr156Met | Missense |  |  | PM2 |  | PM1 PP3 | 4 | |
| *HNF1A* | Exon 2 | c.496T>G | p.Tyr166Asp | Missense |  |  | PM2 |  | PM1 PP3 | 4 | |
| *HNF1A* | Exon 2 | c.514G>T | p.Glu172Ter | Nonsense | PVS1 |  | PM2 |  |  | 5 | |
| *HNF1A* | Exon 2 | c.327-?_526+?del | p.? | Exonic deletion | PVS1 |  | PM2 |  |  | 5 | |
| *HNF1A* | Intron 2 | c.526+1delG | p.? | Splice defect | PVS1 |  | PM2 |  | PP3 | 5 | |
| *HNF1A* | Exon 3 | c.570del | p.Gly191fs | Frameshift | PVS1 |  | PM2 |  |  | 5 | |
| *HNF1A* | Exon 4 | c.775G>A | p.Val259Ile | Missense |  |  | PM2 |  | PM1 PM5 PP3 | 4 | |
| *HNF1A* | Exon 4 | c.825_827del | p.Glu275_Ala276delinsAsp | In-frame deletion | PM4 |  | PM2 |  | PM1 PP3 | 4 | |
| *HNF1A* | Exon 8 | c.1623G>A | p. ? | Splice defect | PVS1 |  | PM2 |  | PP3 | 4 | |
| *HNF1A* | Exon 9 | c.1742_1768+2delinsACAGGG | p.? | Splice defect | PVS1 |  | PM2 |  | PP3 | 4 | |
| *HNF1A* | Exon 9 | c.1697dup | p.His566fs | Frameshift | PVS1 |  | PM2 |  |  | 5 | |
| *HNF1A* | Exon 10 | c.1772_1773del | p.Ser591fs | Frameshift | PS^a^ |  | PM2 |  |  | 4 | |
| *HNF1B* | Exon 1 | c.34C>T | p.Leu12Phe | Missense |  |  | PM2 |  | PM1 PP3 | 4 | |
| *HNF1B* | Exon 2 | c.377A>G | p.Lys126Arg | Missense |  |  | PM2 |  | PM1 PP3 | 4 | |
| *HNF1B* | Exon 2 | c.473C>A | p.Thr158Asn | Missense |  |  | PM2 |  | PM1 PP3 | 4 | |
| *HNF4A* | Exon 4 | c.325C>T | p.Gln109Ter | Nonsense | PVS1 |  |  |  | PM1 | 5 | |
| *HNF4A* | Exon 4 | c.335G>C | p.Arg112Pro | Missense |  |  | PM2 |  | PM5 PP3 | 4 | |
| *HNF4A* | Exon 5 | c.433_436del | p.Ser145fs | Frameshift | PVS1 |  | PM2 |  |  | 5 | |
| *HNF4A* | Exon 5 | c.535T>C | p.Trp179Arg | Missense |  |  | PM2 |  | PM1 PP3 | 4 | |
| *HNF4A* | Exon 6 | c.589del | p.Leu197fs | Frameshift | PVS1 |  | PM2 |  |  | 5 | |
| *HNF4A* | Exon 6 | c.625G>A | p.Gly209Arg | Missense |  |  | PM2 | PP1 | PM1 PP3 | 4 | |
| *HNF4A* | Exon 8 | c.851_852del | p.Gly284fs | Frameshift | PVS1 |  | PM2 | PS2 |  | 5 | |
| *INS* | Exon 2 | c.85C>T | p.His29Tyr | Missense |  |  | PM2 |  | PM5 PP3 | 4 | |
| *INS* | Exon 2 | c.100C>T | p.His34Tyr | Missense |  |  | PM2 |  | PM5 PP3 | 4 | |
| *KCNJ11* | Exon 1 | c.125G>A | p.Cys42Tyr | Missense |  | PS3^b^ | PM2 |  | PM5 PP3 | 4 | |
| *KCNJ11* | Exon 1 | c.952A>G | p.Ile318Val | Missense |  | PS3^b^ |  | PP1 | PP3 | 4 | |

Sequence variants are numbered with respect to GenBank cDNA sequences. *ABCC8*, NM_000352.3; *GCK*, NM_000162.3; *HNF1A*, NM_000545.6; *HNF1B*, NM_000458.3; *HNF4A*, NM_175914.4; *INS*, NM_000207.2; *KCNJ11*, NM_000525.3 and described according to Human Genome Variation Society (HGVS) guidelines (<http://www.hgvs.org/varnomen>). Variants were classified according to ACMG recommendations [1].

**Variant consequence**: PVS1, Nonsense, frameshift, canonical ±1 or ±2 splice sites, single or multi-exon deletions; PS1, same amino-acid change as a previously established pathogenic variant regardless of nucleotide change; PM4, in-frame deletions in a nonrepeat region. ^a^PS instead of PVS1 as this frameshift is located in the last exon of the *HNF1A* gene

**Functional data**: PS3, well-established *in vitro* functional studies supportive of a damaging effect on the gene product; or ^b^improved glycaemic response treated with sulfonylureas.

**Population data**: PM2, if variant allele frequency (VAF) < 0.01% in geographically-matched population databases (ExAC [http://exac.broadinstitute.org/] and dbSNP [www.ncbi.nlm.nih.gov/snp])

**Segregation data**: PS2, *de novo* variant; PP1, segregation with diabetes in at least 2 affected relatives in proband’s family;

**Computational evidence**: PM1, located in a mutational hot spot and/or critical and well-established functional domain; PM5, novel missense change at an amino acid residue where a different missense change determined to be pathogenic has been seen before;

**Predictive evidence:** PP3, *in silico* evidence. For missense mutations, use of 4 predictive algorithms of pathogenicity (SIFT, PolyPhen-2, Align-GVGD and CADD); for CADD, we used a cut-off threshold of 20 (>20: considered pathogenic). For intronic variants, PP3 was attributed if both MaxEntScan and Splice site Finder algorithms predicted a splicing defect according to Houdayer et Coll. [2], guidelines (a variant score at least 15% lower than the reference allele for MaxEntScan and at least 5% lower for Splice site Finder); PP2, Missense variant in a gene that has a low rate of benign missense variation and in which missense variants are a common mechanism of disease.

**Patient’s phenotype**: PP4, proband’s and family’s history was suggestive of monogenic diabetes. Criteria for performing MODY genetic testing were systematically checked before NGS analysis, PP4 was attributed to all patients included in this study.

**Class of pathogenicity** determined taking into account all criteria according to rules established by the ACMG [1].

1. Richards S, Aziz N, Bale S, Bick D, Das S, Gastier-Foster J, Grody WW, Hegde M, Lyon E, Spector E *et al*: **Standards and guidelines for the interpretation of sequence variants: a joint consensus recommendation of the American College of Medical Genetics and Genomics and the Association for Molecular Pathology**. *Genet Med* 2015, **17**(5):405-424.

2. Houdayer C, Caux-Moncoutier V, Krieger S, Barrois M, Bonnet F, Bourdon V, Bronner M, Buisson M, Coulet F, Gaildrat P *et al*: **Guidelines for splicing analysis in molecular diagnosis derived from a set of 327 combined in silico/in vitro studies on BRCA1 and BRCA2 variants**. *Hum Mutat* 2012, **33**(8):1228-1238.

**Table S4.** List of known class 4-5 variants identified in *ABCC8*, *HNF1B*, *KCNJ11* and *INS* genes

| **Gene** | **Location** | **Nucleotide Change** | **Protein effect** | **Variant type** | **References** | **Associated phenotype in literature** |
| --- | --- | --- | --- | --- | --- | --- |
| *ABCC8* | Exon | c.806C>A | p.Ala269Asp | Missense | [1] | NDM |
| *ABCC8* | Exon 28 | c.3547C>T | p.Arg1183Trp | Missense | [2] | TNDM |
| *ABCC8* | Exon 28 | c.3548G>A | p.Arg1183Gln | Missense | [3] | TNDM |
| *ABCC8* | Exon 34 | c.4139G>A | p.Arg1380His (3)^a^ | Missense | [2 ; 4] | TNDM |
| *HNF1B* | Exon 2 | c.529C>T | p.Arg177Ter | Missense | [5] | HNF1B renal disease |
| *HNF1B* | Exon 3 | c.704G>A | p.Arg235Gln | Missense | [7] | HNF1B renal disease |
| *HNF1B* | Exons 1-4 | c.1-?_1045+?del | p.0? (10)^a^ | Exonic deletion | [6] | HNF1B renal disease |
| *HNF1B* | Exons 1-9 | c.1-?_1674+?del | p.0? | Gene deletion | [7] | HNF1B renal disease |
| *KCNJ11* | Exon 1 | c.679G>A | p.Glu227Lys | Missense | [8] | TNDM |
| *INS* | Exon 2 | c.16C>T | p.Arg6Cys (2)^a^ | Missense | [9] | MODY |
| *INS* | Exon 2 | c.130G>A | p.Gly44Arg | Missense | [10]; Bellanné-Chantelot, Saint-Martin unpublished^b^ | MODY |
| *INS* | Exon 3 | c.163C>T | p.Arg55Cys | Missense | [11] | MODY |

Sequence variants are numbered with respect to GenBank cDNA sequences. *ABCC8*, NM_000352.3; *GCK*, NM_000162.3; *HNF1A*, NM_000545.6; *HNF1B*, NM_000458.3; *HNF4A*, NM_175914.4; *INS*, NM_000207.2; *KCNJ11*, NM_000525.3 and described according to Human Genome Variation Society (HGVS) guidelines (<http://www.hgvs.org/varnomen>).

^a^Number of probands into parentheses; ^b^1 additional family diagnosed with TNDM in the diagnostics database of Pitié-Salpêtrière hospital; NDM, neonatal diabetes mellitus; TNDM, transient neonatal diabetes mellitus.

1. Vaxillaire M, Dechaume A, Busiah K, Cave H, Pereira S, Scharfmann R, de Nanclares GP, Castano L, Froguel P, Polak M *et al*: **New ABCC8 mutations in relapsing neonatal diabetes and clinical features**. *Diabetes* 2007, **56**(6):1737-1741.

2. Flanagan SE, Patch AM, Mackay DJ, Edghill EL, Gloyn AL, Robinson D, Shield JP, Temple K, Ellard S, Hattersley AT: **Mutations in ATP-sensitive K+ channel genes cause transient neonatal diabetes and permanent diabetes in childhood or adulthood**. *Diabetes* 2007, **56**(7):1930-1937.

3. Babenko AP, Polak M, Cave H, Busiah K, Czernichow P, Scharfmann R, Bryan J, Aguilar-Bryan L, Vaxillaire M, Froguel P: **Activating mutations in the ABCC8 gene in neonatal diabetes mellitus**. *N Engl J Med* 2006, **355**(5):456-466.

4. Hartemann-Heurtier A, Simon A, Bellanné-Chantelot C, Reynaud R, Cavé H, Polak M, Vaxillaire M, Grimaldi A: **Mutations in the ABCC8 gene can cause autoantibody-negative insulin-dependent diabetes**. *Diabetes Metab* 2009, **35**(3):233-235.

5. Horikawa Y, Iwasaki N, Hara M, Furuta H, Hinokio Y, Cockburn BN, Lindner T, Yamagata K, Ogata M, Tomonaga O *et al*: **Mutation in hepatocyte nuclear factor-1 beta gene (TCF2) associated with MODY**. *Nat Genet* 1997, **17**(4):384-385.

6. Yorifuji T, Fujimaru R, Hosokawa Y, Tamagawa N, Shiozaki M, Aizu K, Jinno K, Maruo Y, Nagasaka H, Tajima T *et al*: **Comprehensive molecular analysis of Japanese patients with pediatric-onset MODY-type diabetes mellitus**. *Pediatr Diabetes* 2012, **13**(1):26-32.

7. Bellanne-Chantelot C, Clauin S, Chauveau D, Collin P, Daumont M, Douillard C, Dubois-Laforgue D, Dusselier L, Gautier JF, Jadoul M *et al*: **Large genomic rearrangements in the hepatocyte nuclear factor-1beta (TCF2) gene are the most frequent cause of maturity-onset diabetes of the young type 5**. *Diabetes* 2005, **54**(11):3126-3132.

8. Edghill EL, Gloyn AL, Goriely A, Harries LW, Flanagan SE, Rankin J, Hattersley AT, Ellard S: **Origin of de novo KCNJ11 mutations and risk of neonatal diabetes for subsequent siblings**. *J Clin Endocrinol Metab* 2007, **92**(5):1773-1777.

9. Edghill EL, Flanagan SE, Patch AM, Boustred C, Parrish A, Shields B, Shepherd MH, Hussain K, Kapoor RR, Malecki M *et al*: **Insulin mutation screening in 1,044 patients with diabetes: mutations in the INS gene are a common cause of neonatal diabetes but a rare cause of diabetes diagnosed in childhood or adulthood**. *Diabetes* 2008, **57**(4):1034-1042.

10. Flannick J, Johansson S, Njolstad PR: **Common and rare forms of diabetes mellitus: towards a continuum of diabetes subtypes**. *Nat Rev Endocrinol* 2016, **12**(7):394-406.

11. Molven A, Ringdal M, Nordbo AM, Raeder H, Stoy J, Lipkind GM, Steiner DF, Philipson LH, Bergmann I, Aarskog D *et al*: **Mutations in the insulin gene can cause MODY and autoantibody-negative type 1 diabetes**. *Diabetes* 2008, **57**(4):1131-1135.

**Table S5.** Main characteristics of the 15 patients with HNF1B-MODY.

| **Patient #** | **HNF1B**  **Nucléotide change / protein effect** | **Sex F/M** | **Eurocaucasian origin yes/no** | **n of generations with diabetes** | **Age at diagnosis of diabetes (years)** | **BMI at diagnosis of diabetes (kg/m²)** | **Symptoms at diagnosis of diabetes^a^ yes/no** | **HbA1c at diagnosis (%)** | **Insulin therapy at diagnosis yes/no** | **Age at study**  **(years)** | **Diabetes duration**  **(years)** | **Plasma creatinine (µmol/L)** | **Renal morphology^b^** |
| --- | --- | --- | --- | --- | --- | --- | --- | --- | --- | --- | --- | --- | --- |
| 1 | c.34C>T / p.Leu12Phe | M | no | 2 | 32 | 24,4 | yes | 10,3 | yes | 33 | 1 | 56 | na |
| 2 | Whole deletion | F | yes | 2 | 15 | 23,4 | yes | na | yes | 18 | 3 | 53 | cysts |
| 3 | Exons 1-4 deletion | M | na | 2 | 18 | na | no | na | no | 66 | 48 | 212 | one cyst |
| 4 | Whole deletion | M | yes | 3 | 27 | 20,2 | yes | 9,9 | yes | 27 | 0 | 88 | normal |
| 5 | Whole deletion | M | no | 3 | 15 | na | yes | na | yes | 25 | 10 | na | normal |
| 6 | Whole deletion | M | no | 3 | 29 | 19,7 | yes | 14 | yes | 29 | 0 | na | na |
| 7 | Whole deletion | M | yes | 1 | 33 | 21,4 | no | 7,1 | no | 35 | 2 | 79 | normal |
| 8 | Whole deletion | M | no | 3 | 34 | 21,8 | yes | 14,2 | yes | 55 | 21 | 98 | normal |
| 9 | Whole deletion | F | yes | 1 | 22 | 19,1 | no | 5,3 | no | 25 | 3 | 61 | cysts |
| 10 | Whole deletion | M | yes | 2 | 18 | na | yes | na | yes | 25 | 7 | 76 | na |
| 11 | c.377A>G / p.Lys126Arg | M | yes | 2 | 39 | 26,1 | yes | na | yes | 45 | 6 | 78 | normal |
| 12 | Whole deletion | M | no | 3 | 22 | 28,5 | yes | 12,1 | yes | 27 | 5 | 89 | normal |
| 13 | c.473C>A / p.Thr158Asn | M | yes | 3 | 15 | 19 | no | 6,6 | no | 16 | 1 | 65 | normal |
| 14 | Whole deletion | F | no | 1 | 40 | 18,4 | yes | 12 | yes | 49 | 9 | 31 | normal |
| 15 | c.704G>A / p.Arg235Gln | F | yes | 3 | 37 | 16,5 | yes | na | yes | 37 | 0 | 54 | na |

^a^Symptoms of diabetes: polyuria and/or unexplained body weight loss and/or diabetic ketoacidosis.

^b^Assessed by ultrasonography and/or computed tomography scan

|  |  |
| --- | --- |

**Table S6.** Main characteristics at the onset of diabetes in patients with monogenic vs. non-monogenic diabetes

|  | **Monogenic** | **Monogenic**  **excluding GCK-cases** | **Non-monogenic^a^** | ***P* ; OR [95% CI]**  **Monogenic vs. non-monogenic** | ***P* ; OR [95% CI]**  **Monogenic excluding GCK**  **vs. non-monogenic** |
| --- | --- | --- | --- | --- | --- |
| N patients | 254^&^ | 142 | 1241 | 254 vs. 1241 | 142 vs. 1241 |
| Sex: F/M | 168/86 (66%) | 89/53 (63%) | 619/622 (50%) | < 10^-4^; 1.96 [1.48-2.60] | 0.0045; 1.69 [1.18-2.41] |
| Eurocaucasian/others | 192/37 (84%) | 101/32 (76%) | 600/499 (55%) | < 10^-4^; 4.32 [2.98-6.26] | < 10^-4^; 2.63 [1.73-3.98] |
| Age (years) | 24 [18-30] (254) | 24 [18.3-29.8] (142) | 31 [25-39] (1239) | < 10^-4^ | < 10^-4^ |
| ≥3 generations with diabetes: yes/no | 144/105 (58%) | 84/55 (60%) | 581/631 (48%) | 0.0053; 1.49 [1.13-1.96] | 0.0055; 1.66 [1.16-2.37] |
| BMI (kg/m²) (n) | 21.8 [20.1-24.2] (221) | 22.8 [20.8-25] (124) | 24.2 [21.6-27.7] (1107) | < 10^-4^ | < 10^-4^ |
| BMI: normal/increased (%) | 180/43 (81%) | 92/33 (74%) | 642/475 (57%) | < 10^-4^; 3.10 [2.18-4.41] | 0.0005; 2.06 [1.36-3.12] |
| Symptoms of diabetes^b^: yes/no | 37/204 (15%) | 33/102 (24%) | 461/702 (40%) | < 10^-4^; 0.28 [0.19-0.40] | 0.0005; 0.49 [0.33-0.74] |
| HbA_1C_ (%) | 6.7 [6.3-8.7] (157) | 8.05 [6.6-9.7] (86) | 9.6 [7-12] (588) | < 10^-4^ | 0.0011 |
| HbA_1C_ (mmol/mol) | 50 [45-72] (157) | 64 [49-83] (86) | 81 [53-108] (588) | < 10^-4^ | 0.0011 |
| Insulin therapy: yes/no | 38/189 (17%) | 36/94 (28%) | 339/768 (31%) | < 10^-4^; 0.46 [0.31-0.66] | 0.5455; 0.87 [0.58-1.30] |
| Hypertension: yes/no | 23/133 (15%) | 16/71 (18%) | 214/538 (28%) | 0.0003; 0.43 [0.27-0.70] | 0.0562; 0.57 [0.32-1.00] |
| Dyslipidemia: yes/no | 23/116 (17%) | 16/63 (20%) | 235/461 (34%) | < 10^-4^; 0.39 [0.24-0.63] | 0.0157; 0.50 [0.28-0.88] |

Values are actual numbers with percentages into parentheses, or median with interquartile range into brackets and numbers of values into parentheses.

^a^Non-monogenic, no genetic etiology detected by targeted NGS on 7 genes;

^b^Symptoms of diabetes: polyuria and/or unexplained body weight loss and/or diabetic ketoacidosis;

BMI, body mass index.

**Table S7.** List of variants of uncertain significance (class 3)

|  |  |  |  | Criteria for classifying variants according to ACMG guidelines [1] | | | | | | |
| --- | --- | --- | --- | --- | --- | --- | --- | --- | --- | --- |
| Gene | Location | Nucleotide Change | Protein effect | Variant type | Variant consequence | Functional data | Population data | Segregation data | Computational and predictive evidence | Class of pathogenicity |
| *ABCC8* | Exon 1 | c.40G>T | p.Ala14Ser | Missense |  |  | PM2 |  | BP4 | 3 |
| *ABCC8* | Exon 3 | c.291G>T | p.? | Splice defect |  |  | PM2 |  |  | 3 |
| *ABCC8* | Exon 3 | c.361G>A | p.Val121Met | Missense |  |  | PM2 |  | PP3 | 3 |
| *ABCC8* | Exon 3 | c.380T>C | p.Ile127Thr | Missense |  |  | PM2 |  | PP3 | 3 |
| *ABCC8* | Intron 4 | c.580-16_580-14del | p.? | Splice defect |  |  |  |  |  | 3 |
| *ABCC8* | Exon 5 | c.770T>C | p.Met257Thr | Missense |  |  | PM2 |  |  | 3 |
| *ABCC8* | Exon 5 | c.809T>G | p.Phe270Cys | Missense |  |  | PM2 |  | PP3 | 3 |
| *ABCC8* | Exon 6 | c.946G>A^a^ | p.Gly316Arg | Missense |  |  | PM2 |  | PP3 | 3^b^ |
| *ABCC8* | Exon 9 | c.1337T>C | p.Ile446Thr | Missense |  |  |  |  | PP3 | 3 |
| *ABCC8* | Exon 10 | c.1510C>T | p.Arg504Cys | Missense |  |  |  | BP5 | PP3 | 3 |
| *ABCC8* | Exon 10 | c.1537G>A | p.Ala513Thr | Missense |  |  | PM2 |  | PP3 | 3^b^ |
| *ABCC8* | Exon 15 | c.2104C>T | p.Arg702Cys | Missense |  |  | PM2 |  | PP3 | 3 |
| *ABCC8* | Exon 15 | c.2116+61A>G | p.? | Splice defect |  |  | PM2 |  | PP3 | 3 |
| *ABCC8* | Exon 21 | c.2491T>A | p.Ser831Thr | Missense |  |  | PM2 |  | PP3 | 3 |
| *ABCC8* | Exon 21 | c.2512A>G | p.Ile838Val | Missense |  |  | PM2 |  |  | 3 |
| *ABCC8* | Exon 21 | c.2546A>C | p.Asn849Thr | Missense |  |  | PM2 |  | PP3 | 3 |
| *ABCC8* | Exon 25 | c.3113C>A | p.Thr1038Asn | Missense |  |  | PM2 |  | PP3 | 3 |
| *ABCC8* | Exon 28 | c.3496G>A | p.Val1166Met | Missense |  |  | PM2 |  | PP3 | 3 |
| *ABCC8* | Exon 31 | c.3778G>A | p.Val1260Met | Missense |  |  | BS1 |  | PP3 | 3 |
| *ABCC8* | Exon 31 | c.3827T>C | p.Leu1276Pro | Missense |  |  | PM2 |  | PP3 | 3 |
| *ABCC8* | Exon 31 | c.3868A>G | p.Met1290Val | Missense |  |  | PM2 |  | BP4 | 3 |
| *ABCC8* | Exon 35 | c.4279C>A | p.Gln1427Lys | Missense |  |  | PM2 |  | PP3 | 3 |
| *ABCC8* | Exon 37 | c.4511T>A | p.Ile1504Asn | Missense |  |  | PM2 |  | PP3 | 3 |
| *ABCC8* | Exon 39 | c.4736G>A | p.Arg1579His | Missense |  |  | PM2 |  | PP3 | 3 |
| *GCK* | Exon 2 | c.142G>A | p.Glu48Lys | Missense |  |  | PM2 |  | PP3 PP2 | 3 |
| *GCK* | Exon 3 | c.325T>C | p.Ser109Pro | Missense |  |  | PM2 |  | PP3 PP2 | 3 |
| *GCK* | Intron 5 | c.580-9T>G^†^ | p.? | Splice defect |  |  | PM2 |  | PP3 | 3 |
| *GCK* | Exon 8 | c.923G>C | p.Arg308Thr | Missense |  |  | PM2 |  | PP3 PP2 | 3 |
| *GCK* | Exon 9 | c.1207C>T | p.Arg403Cys | Missense |  |  | PM2 |  | PP3 PP2 | 3 |
| *GCK* | Exon 10 | c.1373_1376del^†^ | p.Lys458fs | Frameshift | PM4 |  | PM2 |  |  | 3 |
| *HNF1A* | Promoter | c.-191T>C | p.= | Promoter variant |  |  | PM2 |  |  | 3 |
| *HNF1A* | Exon 1 | c.98C>T | p.Pro33Leu | Missense |  |  | PM2 | PP5 | BP4 | 3 |
| *HNF1A* | Exon 2 | c.490A>G | p.Thr164Ala | Missense |  |  | PM2 | PP5 | PM1 BP4 | 3 |
| *HNF1A* | Exon 2 | c.511C>G | p.Arg171Gly | Missense |  |  | PM2 | PP5 | PM1 BP4 | 3^b^ |
| *HNF1A* | Exon 4 | c.866C>T | p.Pro289Leu | Missense |  |  | PM2 |  | PP3 | 3 |
| *HNF1A* | Exon 7 | c.1380_1406del | p.Gln460_Leu468del | In-frame deletion | PM4 |  |  |  |  | 3 |
| *HNF1A* | Exon 8 | c.1522G>A | p.Glu508Lys | Missense |  | PS3 |  |  | PP3 | 3^b^ |
| *HNF1A* | Exon 10 | c.1865T>C | p.Ile622Thr | Missense |  |  | PM2 |  | PP3 | 3 |
| *HNF1B* | Exon 1 | c.118G>A | p.Gly40Arg | Missense |  |  | PM2 |  | PP3 | 3 |
| *HNF1B* | Exon 7 | c.1460T>C | p.Met487Thr | Missense |  |  | PM2 |  | PP3 | 3 |
| *HNF1B* | Exon 7 | c.1484T>A | p.Met495Lys | Missense |  |  | PM2 |  | PP3 | 3 |
| *HNF4A* | Exon 2 | c.203A>G | p.Lys68Arg | Missense |  |  | PM2 |  | PP3 | 3 |
| *HNF4A* | Exon 4 | c.353G>A | p.Arg118Gln | Missense |  |  | PM2 |  | PP3 | 3 |
| *HNF4A* | Exon 5 | c.461T>G | p.Ile154Ser | Missense |  |  | PM2 |  | PP3 | 3 |
| *HNF4A* | Exon 5 | c.478G>A | p.Ala160Thr | Missense |  |  | PM2 |  | PP3 | 3 |
| *HNF4A* | Exon 6 | c.658G>A | p.Val220Met | Missense |  |  | PM2 |  | PP3 | 3^b^ |
| *INS* | Exon 2 | c.11G>A | p.Trp4Ter | Nonsense |  |  | PM2 |  | PP3 | 3^c^ |
| *INS* | Exon 2 | c.155C>T | p.Pro52Leu | Missense |  |  | PM2 |  | PP3 | 3 |
| *INS* | Intron 2 | c.188-15G>A | p.? | Splice defect |  |  | PM2 |  | PP3 | 3^b^ |
| *KCNJ11* | Exon 1 | c.86G>A | p.Arg29His | Missense |  |  | PM2 |  | PP3 | 3 |
| *KCNJ11* | Exon 1 | c.160C>T | p.Arg54Cys | Missense |  |  | PM2 |  | PP3 | 3 |
| *KCNJ11* | Exon 1 | c.341T>C | p.Ile114Thr | Missense |  |  | PM2 |  | PP3 | 3 |
| *KCNJ11* | Exon 1 | c.353C>T | p.Ser118Leu | Missense |  |  | PM2 |  | BP4 | 3^b^ |
| *KCNJ11* | Exon 1 | c.463G>A | p.Val155Met | Missense |  |  | PM2 |  | PP3 | 3 |
| *KCNJ11* | Exon 1 | c.623G>A | p.Ser208Asn | Missense |  |  | PM2 |  | PP3 | 3 |
| *KCNJ11* | Exon 1 | c.662G>A | c.Arg221His | Missense |  |  | PM2 |  | PP3 | 3 |

^a^Variant identified in two probands; ^b^Variant previously reported in literature; ^c^Variant identified at an heterozygous state and previously reported in literature at homozygous state in type 1-like diabetes [2].

Sequence variants are numbered with respect to GenBank cDNA sequences. *ABCC8*, NM_000352.3; *GCK*, NM_000162.3; *HNF1A*, NM_000545.6; *HNF1B*, NM_000458.3; *HNF4A*, NM_175914.4; *INS*, NM_000207.2; *KCNJ11*, NM_000525.3 and described according to Human Genome Variation Society (HGVS) guidelines [3].

Variant evaluation was conducted as described in **Supplemental Table S2**. One additional criterion was considered for these variants as piece of predictive evidence : BP4, when results of prediction algorithm are contradictory and do not suggest any impact on the gene product.

1. Richards S, Aziz N, Bale S, Bick D, Das S, Gastier-Foster J, Grody WW, Hegde M, Lyon E, Spector E *et al*: **Standards and guidelines for the interpretation of sequence variants: a joint consensus recommendation of the American College of Medical Genetics and Genomics and the Association for Molecular Pathology**. *Genet Med* 2015, **17**(5):405-424.

2. Di Benedetto M, Richard O, Pélissier P, Darteyre S, Cavé H, Stéphan JL: **[Permanent neonatal diabetes and recessive mutation in the INS gene: a familial history]**. *Arch Pediatr* 2013, **20**(2):199-202.

3. den Dunnen JT, Dalgleish R, Maglott DR, Hart RK, Greenblatt MS, McGowan-Jordan J, Roux AF, Smith T, Antonarakis SE, Taschner PE: **HGVS Recommendations for the Description of Sequence Variants: 2016 Update**. *Hum Mutat* 2016, **37**(6):564-569.

**Figure S1.** Main characteristics at diagnosis of diabetes in patients with monogenic diabetes according to the involved gene

**
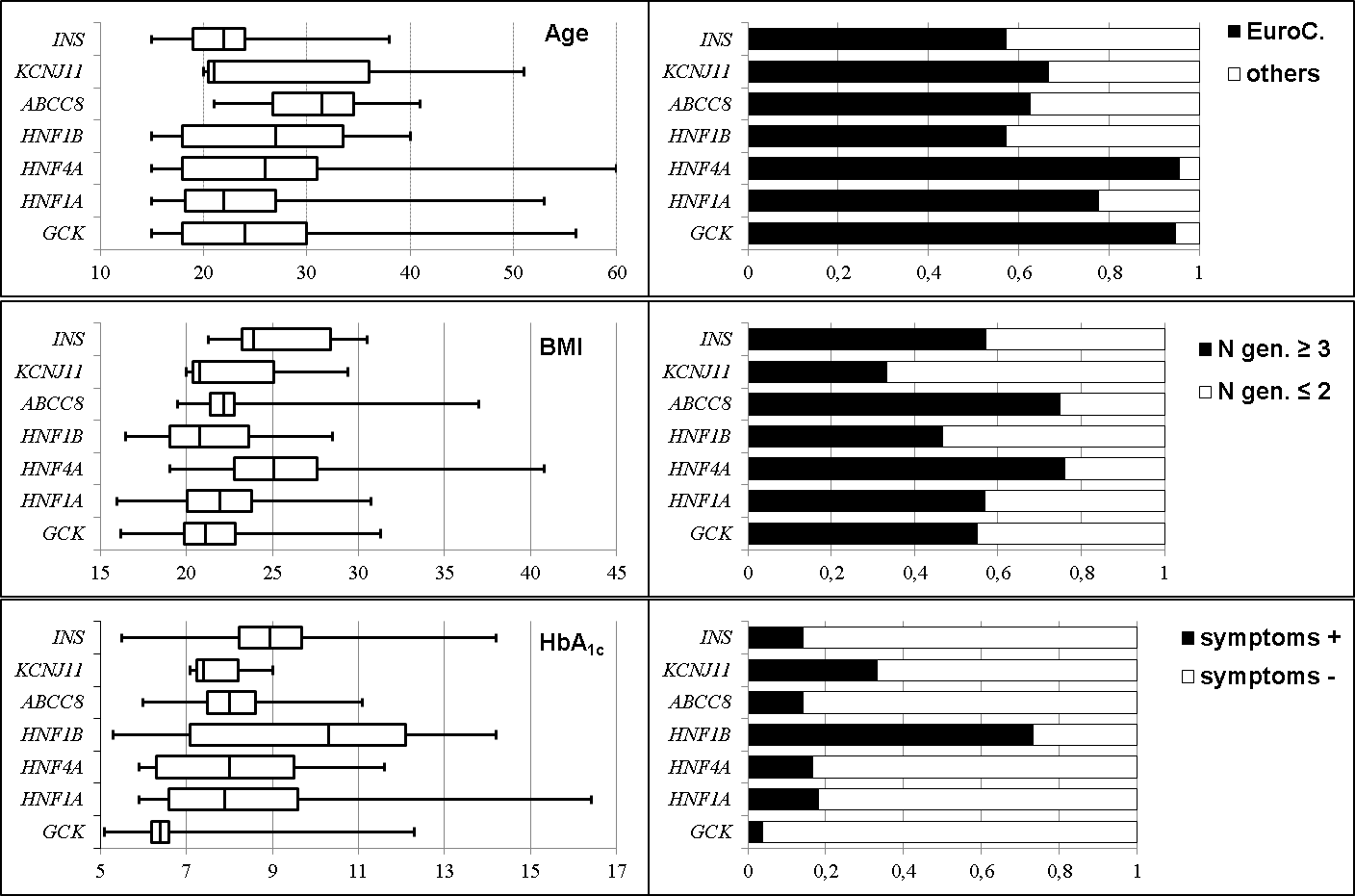
**

Legend. Box-plot representation of age (years), BMI, body mass index (kg/m²), HbA_1c_ (%), and percentages of geographical origin family history of diabetes in ≥ 3 generations, and presence of diabetes symptoms.

**Figure S2.** Main characteristics at diagnosis of diabetes in patients with (M+) and without (M-) monogenic diabetes

**
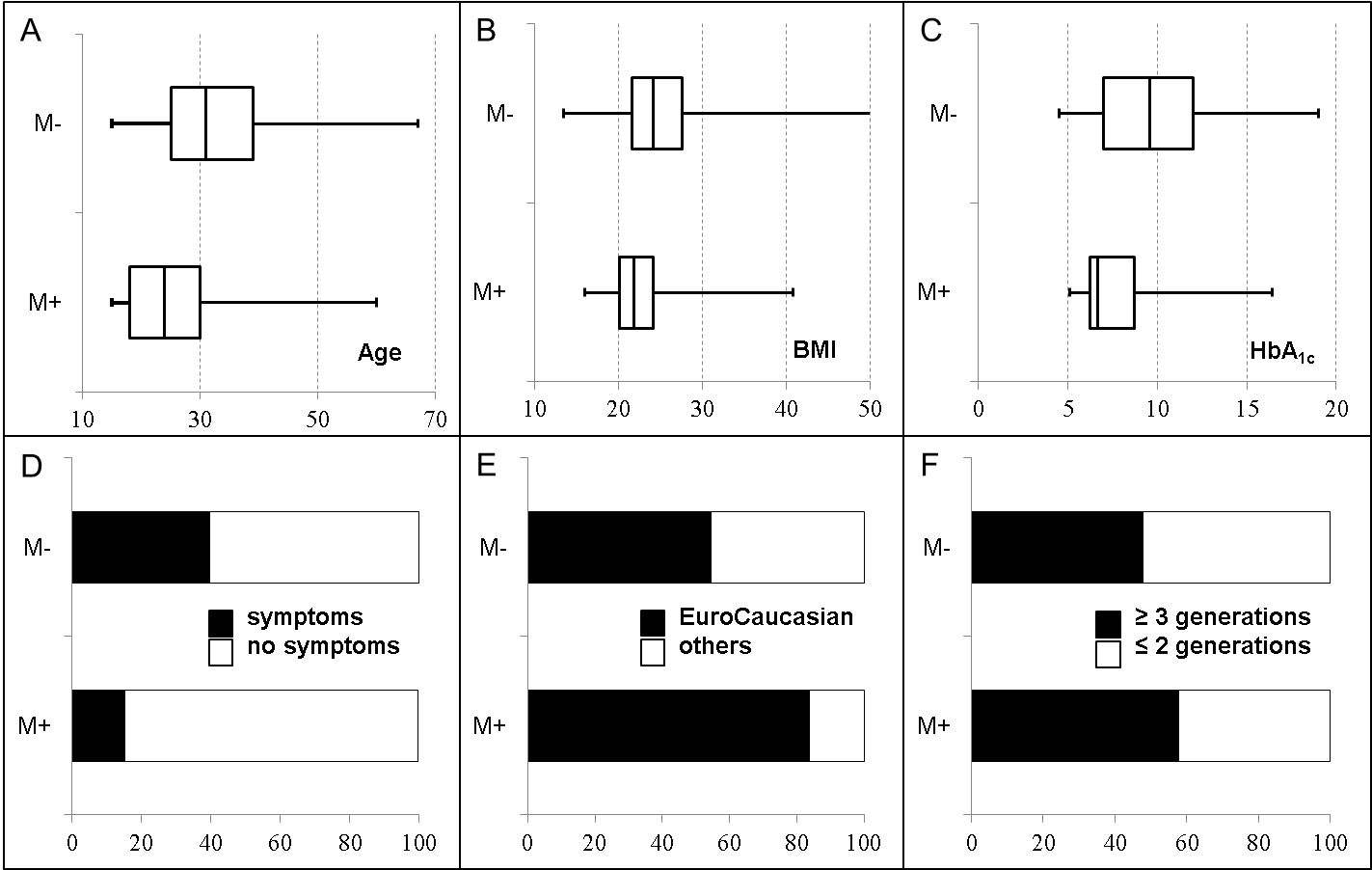
**

Legend. Box-plot representation of A, age (years), B, BMI, body mass index (kg/m²), C, HbA_1c_ (%), and percentages of D, presence of

diabetes symptoms, E, geographical origin, and F, family history of diabetes in ≥3 generations.

**Figure S3.** Hierarchical clustering of 1495 patients with a clinical suspicion of monogenic diabetes


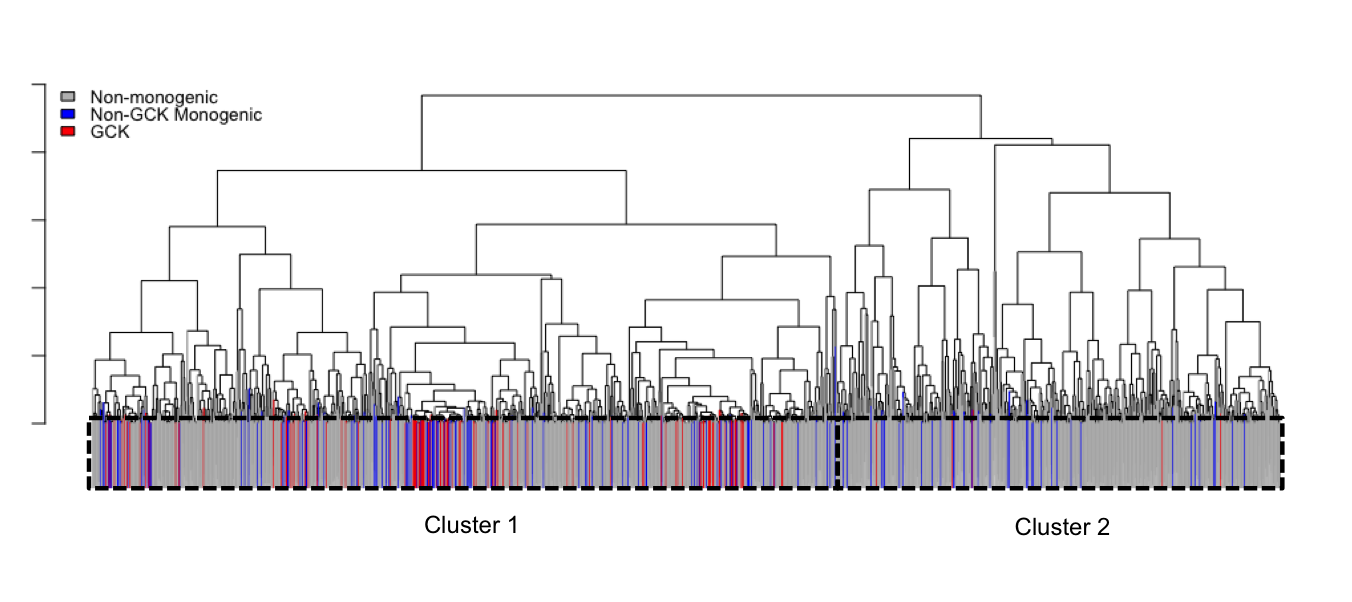


Legend. Dendrogram of the non-supervised hierarchical clustering performed in 1495 patients with a clinical suspicion of monogenic diabetes. Patients with loss-of-function variants (n=10) and those with a class 3 variant (n=59) were not included in this analysis.
